# A Review of Pedestrian Trajectory Prediction Methods Based on Deep Learning Technology

**DOI:** 10.3390/s25237360

**Published:** 2025-12-03

**Authors:** Xiang Gu, Chao Li, Long Gao, Xuefen Niu

**Affiliations:** 1 Yongyou School, Nantong Institute of Technology, Nantong 226001, China; gu.x@ntu.edu.cn; 2 School of Artificial Intelligence and Computer Science, Nantong University, Nantong 226001, China; 2330320066@stmail.ntu.edu.cn; 3 Jiangsu Datang International Lüsigang Power Generation Co., Ltd., Lüsigang, Nantong 226200, China; 4 School of Mathematics and Statistics, Nantong University, Nantong 226001, China; niuxuefen@tkgjhr.com; 5 Suzhou Lanjixing Human Resources Services Co., Ltd., Suzhou 215000, China

**Keywords:** pedestrian trajectory prediction, review, autonomous driving, deep learning

## Abstract

Pedestrian trajectory prediction is a critical component of autonomous driving and intelligent urban systems, with deep learning now dominating the field by overcoming the limitations of traditional models in handling multi-modal behaviors and complex social interactions. This survey provides a systematic review and critical analysis of deep learning-based approaches, offering a structured examination of four key model families: RNNs, GANs, GCNs, and Transformer. Unlike previous reviews, we introduce a comparative analytical framework that evaluates each method’s strengths and limitations across standardized criteria. The review also presents a comprehensive taxonomy of datasets and evaluation metrics, highlighting both established practices and emerging trends. Finally, we derive future research directions directly from our critical assessment, focusing on semantic scene understanding, model transferability, and the precision–efficiency trade-off. Our work provides both a historical perspective on methodological evolution and a forward-looking analysis to guide future research development.

## 1. Introduction

In recent years, technological advancements have driven the development of automation and intelligent systems, leading to the emergence of an increasing number of autonomous solutions. Among these, trajectory prediction has become a key focus in social robotics [1,2,3,4,5,6], autonomous driving [7], and video surveillance. Furthermore, according to a report by the World Health Organization, approximately 1.35 million people die in road traffic accidents each year, with pedestrians accounting for 23% of the fatalities [8]. These accidents often occur at intersections with low visibility and crowded spaces, where drivers’ attention is diminished and pedestrians lack any protective measures. Therefore, reducing these risks and ensuring road safety is a critical issue. Under these conditions, pedestrian trajectory prediction can help enhance the safety of road users by assisting drivers in reducing or avoiding human errors such as fatigue and distraction, as well as enabling autonomous systems to anticipate pedestrian motion. Pedestrians and cyclists are among the most vulnerable road users. Understanding their intended trajectories enables autonomous vehicles to take proactive measures to prevent collisions. Socially aware service robots optimize their navigation by predicting and recognizing pedestrian trajectories and intentions [9,10,11], thereby avoiding unnecessary contact. Similarly, in tracking and video surveillance systems, pedestrian movements are predicted based on their historical trajectories and interactions with both other pedestrians and the surrounding environment. As a result, pedestrian trajectory prediction has become increasingly crucial across various applications, including autonomous vehicles, social robotics, and surveillance systems. Predicting pedestrian trajectories is a highly challenging task due to the unpredictable nature of human movement, which can change dynamically based on interactions with objects, vehicles, and other pedestrians. Achieving precise trajectory predictions is inherently difficult. Ideally, trajectory forecasting would be based on known pedestrian destinations. However, in real-world scenarios, pedestrian destinations are often unknown, and their movements are influenced not only by their own intentions but also by the behavior of those around them. Moreover, traditional pedestrian trajectory prediction models typically function as input-to-output mappings. Most modern approaches rely on neural networks that model sequence-to-sequence relationships, often generating predictions based on the average predicted trajectory. This frequently results in compromised trajectory estimations that lack accuracy and realism.This paper aims to provide a comprehensive review and comparison of recent deep learning-based approaches for pedestrian trajectory prediction. The key contributions of this paper are as follows:

- A comprehensive and multi-faceted review of deep learning-based pedestrian trajectory prediction models.
- A systematic categorization and summary of datasets and evaluation metrics used in pedestrian trajectory prediction.
- Insights into future research directions and key challenges in the field.

The remainder of this paper is organized as follows: Section 2 reviews related work on pedestrian trajectory prediction, including problem definitions and methodological developments. Section 3 categorizes and analyzes existing trajectory prediction approaches. Section 4 discusses multi-modal trajectory prediction. Section 5 provides a comprehensive summary and critical analysis of pedestrian trajectory prediction methods. Section 6 introduces widely used public datasets and evaluation metrics. Section 7 explores current challenges and future research directions. Finally, Section 8 presents the conclusions of this paper.

Distinguishing from the methodological enumeration structure of existing surveys, this paper introduces a ‘Socio-Cognitive and Technical Implementation’ dual-layer analytical framework as our organizational axis. At the socio-cognitive layer, we conceptualize pedestrian trajectory prediction as inference of human intentions and decision processes, emphasizing the influences of social norms, environmental semantics, and individual differences. At the technical implementation layer, we analyze how model architectures transform these cognitive elements into computational representations. This framework reveals the core tension in the field’s development: increased technical complexity does not linearly correlate with enhanced cognitive authenticity. For instance, complex Transformer models may outperform simple LSTMs on ETH/UCY datasets, yet in real urban scenarios, hybrid architectures incorporating social force prior knowledge often demonstrate superior robustness. The dual-layer framework not only provides a systematic knowledge organization method but also establishes a new standard for evaluating the essential value of methods: technical merit should not be measured solely by ADE/FDE metrics, but more importantly by a model’s capacity to respect and express fundamental patterns of human behavior.

## 2. Related Work

### 2.1. Description of the Pedestrian Trajectory Prediction Problem

In trajectory prediction, an agent refers to a self-aware road user, such as a pedestrian, cyclist, or driver. The fundamental objective of trajectory prediction is to infer the agent’s potential future states based on its current state and surrounding environmental factors. This task involves leveraging positional and self-state information to predict future trajectory coordinates using historical movement data. The core challenge in pedestrian trajectory prediction lies in utilizing observed historical trajectories to develop models that can learn interactions among pedestrians, between pedestrians and objects, and between pedestrians and their environment, thereby enabling short-term future movement predictions. Rudenko et al. [12] provide a foundational taxonomy based on two key aspects: the motion model and input contextual cues, covering applications in service robotics, autonomous vehicles, and surveillance systems.

Generally, the problem can be structured into three fundamental components: data acquisition and processing, modeling approaches, and prediction output formats. Pedestrian movement is influenced by both individual intentions and environmental factors. By analyzing historical trajectories, key kinematic features—such as position, velocity, and body movement—as well as static and dynamic environmental interactions can be captured to enhance prediction accuracy. In practical applications, sensor technologies including radio detection, light detection, LiDAR, and cameras are widely employed to support this task. Different modeling techniques significantly impact prediction performance. Traditional approaches often rely on parameterized models grounded in motion dynamics, such as the social force model [9] and hybrid estimation methods, which offer valuable insights into pedestrian interactions. However, such models tend to be limited to specific scenarios due to their inability to fully capture complex interactive behaviors, thereby constraining their generalizability. In contrast, modern trajectory prediction methods increasingly adopt pattern-based and data-driven techniques [13], which learn to approximate complex motion functions and exhibit substantially improved generalization across diverse environments.In terms of prediction output, the trajectory of an agent *i* is typically represented as a sequence of 2D or pixel coordinates in real-world space [14]: $X_{i}^{T},Y_{i}^{T}$, where $X_{i}^{T}=X_{i}^{t}|t\in \left[1,T_{\mathrm{obs}}\right]$ denotes the observed trajectory over $T_{\mathrm{obs}}$ time steps, and $Y_{i}^{T}=Y_{i}^{t}|t\in \left[T_{\mathrm{obs}},T_{\mathrm{obs}}+T_{\mathrm{pred}}\right]$ represents the ground-truth future path over $T_{\mathrm{pred}}$ steps. The objective of trajectory prediction is to optimize a model that generates future trajectories conditioned on observed information $X_{i}^{T}$, neighboring agent trajectories $X_{\left(i:N/i\right)}^{T}$, and scene context *S*. When the model is constrained to predict a single future trajectory per agent, the task is termed deterministic trajectory prediction. Conversely, if multiple plausible future trajectories are generated, it is categorized as multi-modal trajectory prediction.

### 2.2. Introduction of the Main Methods of Pedestrian Trajectory Prediction

**Figure 1:**
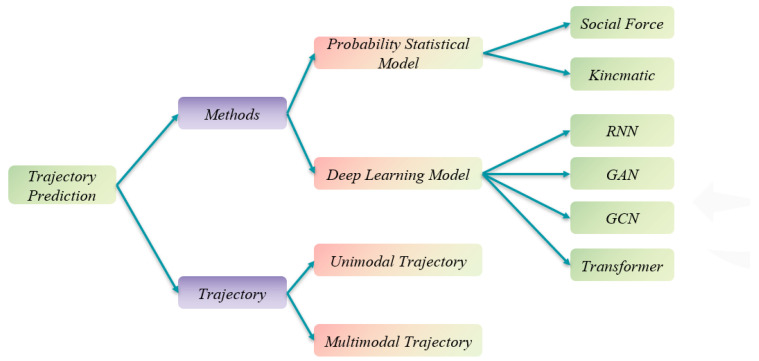
Classification of pedestrian trajectory prediction methods.

The earliest methods for pedestrian trajectory prediction were based on statistical models. However, due to the lack of standardized evaluation metrics, the assessment results of these models were often non-comparable, complicating the task of selecting the most appropriate method. Additionally, the datasets used were often sparse and incomplete, further limiting the practical applicability of these models. With the advent of artificial intelligence, deep learning has progressively been integrated into trajectory prediction, leading to a shift in modeling approaches. Consequently, current methods are predominantly classified into two main categories, as illustrated in Figure 1. This section provides a brief overview of each model, categorized by differences in their modeling approaches. In the early stages of trajectory prediction research, statistical models were commonly employed. These methods combined basic kinematic models with techniques such as Bayesian filters [15], Markov networks [16,17], and Bayesian networks to predict future state information from current state data. When utilizing Bayesian filters for model interaction, the assumption of constant speed does not accurately reflect real-world dynamics. Moreover, these models struggle to capture the dynamic state information of pedestrians. The switched linear dynamic system (SLDS) model [18], which uses a Markov chain for state switching, faces challenges when dealing with more complex motion patterns, as the available motion features are insufficient for effective state transitions.

#### 2.2.1. Introduction of Trajectory Prediction Method Based on Probability Statistical Models

The dynamic Bayesian network model for pedestrian path prediction [19] provides more accurate predictions than the SLDS model by incorporating contextual information. Hebing et al. [9] introduced the social force model, which is widely applied in path selection and activity understanding, as it captures the interaction information between individuals [10,11]. With the rapid development of machine learning, tracking algorithms such as the Kalman filter [20], linear regression [21], Markov models, Gaussian processes [22,23,24,25,26], autoregressive models [27], and time series analysis [28] have been utilized to enhance trajectory prediction. However, these methods still rely heavily on kinematic models and often require prior assumptions. While effective for conventional problems, their predictive accuracy may significantly deviate from real-world behavior when applied to more complex scenarios. These probabilistic statistical models, while structurally simple, are highly sensitive to parameters and exhibit poor generalization ability. Furthermore, the process of predictive reasoning and model development often involves numerous assumptions and calculations, making it challenging to apply these models effectively to specific real-world scenarios, such as sidewalks or intersections. As a result, there remains a substantial gap between their predictions and actual pedestrian behavior.

#### 2.2.2. Introduction to Trajectory Prediction Methods Based on Deep Learning Models

**Figure 2:**
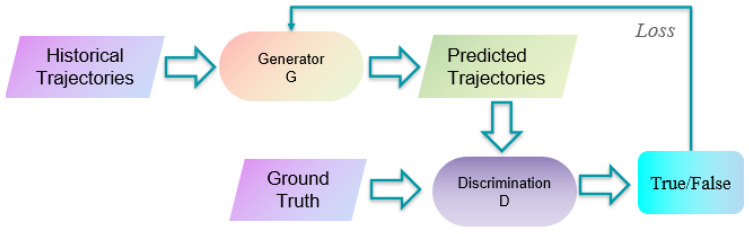
GANs architecture for pedestrian trajectory prediction.

Pedestrian trajectory prediction based on recurrent neural networks (RNNs) [29] is a widely used technology in computer vision. RNNs are specifically designed to process sequential data, making them highly effective for time series or time-dependent tasks. They have achieved significant success in fields such as natural language processing and sequence prediction, including applications like speech recognition [30,31,32,33], caption generation [34,35,36,37,38], machine translation [39,40,41], and semantic segmentation [42]. Additionally, RNNs have been applied to image classification [43,44,45,46] and scene analysis [47]. In pedestrian trajectory prediction, RNNs predict future movements by learning patterns and contextual information from historical trajectory data. These models incrementally predict future trajectory points based on past trajectory data. A common approach involves using long short-term memory (LSTM) networks [48] or gated recurrent units (GRUs) [49] as the core components of RNNs. These units are especially effective at capturing long-term dependencies within sequences by adaptively updating and maintaining an internal memory state.Numerous methods in pedestrian trajectory prediction focus on RNNs, with significant success in the field. However, RNN models have limitations. Traditional RNNs struggle with the vanishing or exploding gradient problem when dealing with long-term dependencies, making it difficult to capture relevant information over extended time periods. Additionally, as autoregressive models, multi-step predictions tend to accumulate errors, causing predicted trajectories to deviate from actual outcomes. RNNs also face challenges in handling variable-length sequence data, and their training complexity, combined with large data requirements, can hinder the model’s generalization ability. In recent years, generative adversarial networks (GANs) [50] have emerged as a prominent approach for pedestrian trajectory prediction. Since pedestrian trajectory distributions exhibit multi-modal characteristics, GAN-based models can learn the data distribution and generate new samples, overcoming the limitation of RNNs, which typically output a single average prediction, failing to account for the full range of possible future trajectories. GANs consist of two networks: a generator and a discriminator. As shown in Figure 2, the generator takes the current pedestrian trajectory as input and attempts to generate future trajectories, while the discriminator evaluates the generated trajectories by distinguishing them from real trajectories. During training, the generator and discriminator engage in a competitive process. The generator improves its predictions by minimizing the difference between its generated trajectories and real ones, while the discriminator works to maximize its ability to distinguish between generated and real trajectories. In addition to GANs, a popular alternative is the variational autoencoder (VAE) [51], which consists of two components: an encoder and a decoder. By introducing the concept of variational inference, VAEs can generate samples with some degree of diversity. However, VAEs generally struggle to model deep interactions between individuals. The uncertainty, diversity, and long-term dependencies in pedestrian trajectory data, along with the complex assumptions about the latent variable distribution in VAEs, complicate probability calculations. Compared to VAEs, GANs do not require explicitly designed encoder and decoder networks for generating trajectory samples, simplifying the model architecture and training process. Moreover, GANs can generate predictions that capture contextual relationships, allowing them to better model long-term dependencies in pedestrian trajectories. Through adversarial training, GANs can produce more realistic and diverse trajectory samples, ultimately improving prediction accuracy. However, GANs also face challenges such as training instability and mode collapse.

**Figure 3:**
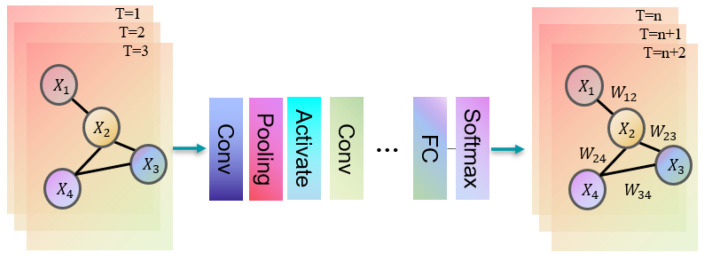
Graph neural network architecture for pedestrian trajectory prediction.

**Figure 4:**
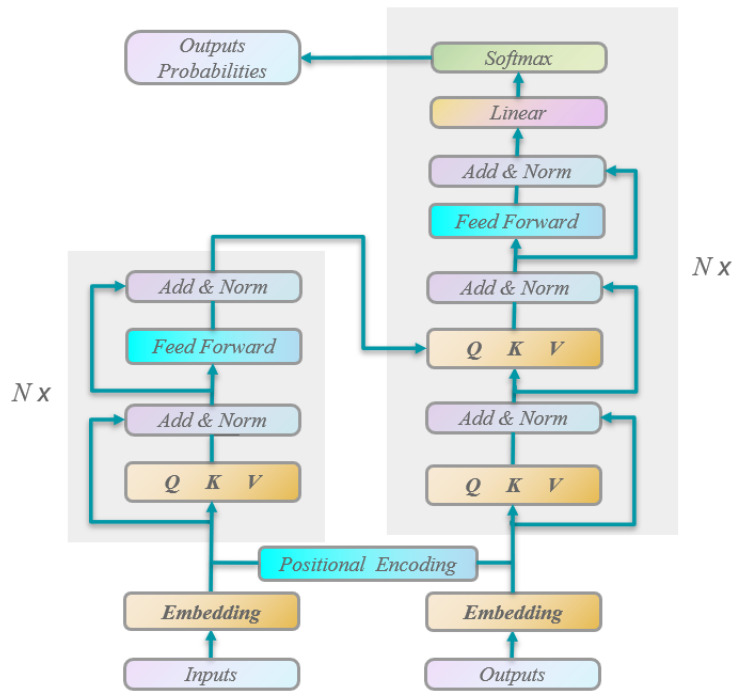
Transformer architecture for pedestrian trajectory prediction.

Pedestrian trajectory prediction using graph convolutional networks (GCNs) [52] leverages GCNs to model the relationships between pedestrians and predict their future trajectories by learning from a graph structure. This graph-based approach intuitively represents the interactions between pedestrians, addressing the spatial modeling limitations of conventional RNN models. It is also more effective than methods that aggregate RNN outputs. The core concept of GCNs is to model and learn the spatial relationships between pedestrians by constructing a pedestrian interaction graph using graph convolution operations, as illustrated in Figure 3. This framework allows GCNs to capture dependencies and contextual relationships between pedestrians more effectively, leading to more accurate and realistic trajectory predictions. Many approaches incorporate graph structures, with spatio-temporal graph models being a common framework. These models predict future pedestrian trajectories by capturing both spatial and temporal relationships, often combining GCNs with RNNs or other sequence models to account for temporal dependencies. By modeling spatial relationships, GCN-based methods offer comprehensive information for trajectory prediction, demonstrating strong robustness and generalization capabilities. However, determining the precise connections between pedestrians in complex interaction environments remains challenging. Additionally, as the graph size increases, the computational complexity also rises, making scalability a concern in more intricate scenarios. Pedestrian trajectory prediction based on the Transformer model [53] employs the Transformer architecture to capture spatio-temporal relationships in pedestrian trajectories and learn contextual information for prediction. The model consists of an encoder and a decoder, as shown in Figure 4. The encoder captures the spatio-temporal relationships in pedestrian trajectories, while the decoder generates future trajectory predictions. The Transformer leverages its self-attention mechanism to facilitate information association and redistribution across different positions in the input sequence. Compared to traditional RNNs, the Transformer is more effective at capturing long-range dependencies. Additionally, the model utilizes a multi-layer stacked encoder structure, which enables it to leverage information from the entire sequence and improve prediction accuracy. However, when handling long sequences, the Transformer may encounter challenges, such as reduced prediction accuracy and high computational resource consumption, due to the increased computational complexity introduced by the self-attention mechanism. In recent years, numerous deep learning-based methods have emerged in the relevant literature. This section provides a detailed introduction to the most commonly used approaches in this field, categorizing them based on the structural types of deep neural networks (DNNs).

## 3. Analysis and Comparison of Pedestrian Trajectory Prediction Methods

Through the lens of our ‘Socio-Cognitive and Technical Implementation’ dual-layer framework, we identify that the four mainstream method categories (RNNs, GANs, GCNs, Transformers) embody distinct philosophical orientations: RNN models implicitly adopt a ‘behavioral inertia’ hypothesis, assuming future trajectories are primarily determined by historical movement patterns; GANs embody a ‘social diversity’ philosophy, emphasizing the multi-modal nature of human behavior; GCNs reflect a ‘spatial interaction centrism,’ treating social influence as information flow on graph structures; while Transformers represent a ‘global context awareness’ paradigm, asserting that prediction requires integration of relationships among all elements in the scene. This insight transcends superficial ‘architecture comparison’ analysis, revealing that method selection essentially constitutes a choice of fundamental assumptions about human behavior. Our evaluation demonstrates that state-of-the-art methods are often not those with the most complex architectures, but rather models achieving optimal balance between socio-cognitive principles and technical implementation—they respect the social essence of human behavior while possessing sufficient expressive capacity to capture this complexity. This observation leads us to propose the ‘Cognition-Technology Alignment Principle’: optimal prediction models should match the social complexity of their application environments, rather than pursuing architectural advancement indiscriminately.

### 3.1. Analysis and Comparison of Methods Based on RNN Model

Long Short-Term Memory (LSTM) networks have effectively solved the problem of long-term information retention, making them a key tool in sequence prediction tasks. LSTM has achieved notable success in various fields, including pedestrian trajectory prediction. For example, some researchers have applied the LSTM model to learn patterns of human activity and environmental factors. Similarly, Others have used an RNN and encoder-decoder architecture to analyze full-body posture and predict pedestrian future positions. While LSTM excels at learning from long sequences, it still struggles to capture dependencies among multiple related sequences. Alahi et al. [14] introduced the S-LSTM model, which incorporates a “social” component to predict pedestrian dynamics in continuous space. This model uses a social pooling layer to share hidden state information among pedestrians. It was the first RNN-based approach to simulate human-to-human interactions and outperformed traditional statistical models on the ETH and UCY datasets. To better manage temporal and spatial contexts, Liu et al. [54] proposed a spatio-temporal recurrent neural network (ST-RNN). This model predicts future pedestrian behavior by employing a transfer matrix to capture local temporal and spatial contexts across varying time intervals and geographic distances. Lee et al. [55] developed the DESIRE model, a deep stochastic inverse optimal control RNN encoder–decoder framework. This model uses a variational autoencoder to generate prediction samples, which are refined using an RNN. The integration of dynamic and static information, scene context, and agent interactions improves long-term prediction accuracy. The model was tested on the KITTI and Stanford University UAV datasets. Su et al. [56] proposed a social-aware LSTM model, which embeds prior knowledge into the data using a recursive Gaussian process to explore crowd dynamics. Their model is effective even in crowded environments where trajectories are occluded or unpredictable. MX-LSTM [57] is an LSTM architecture that integrates location and orientation information. By optimizing LSTM parameters with Gaussian full covariance, it connects human head motion data with position information, providing good prediction performance, even at lower speeds. Vemula et al. [58] introduced a trajectory prediction model called social attention (S-attention), which uses a spatio-temporal graph to model positional relationships among pedestrians. It calculates the soft attention of neighboring RNN hidden states to capture the relative importance of each pedestrian in a crowd. Femado et al. [59] proposed a model combining a soft attention mechanism with a hard-wired approach to map trajectory information to future positions. This model, based on an LSTM encoder–decoder architecture, not only predicts trajectories but also detects abnormal behaviors. Xue et al. [60] developed a hierarchical LSTM network, named Social-Scene-LSTM, to account for scene layout in pedestrian trajectory prediction. This model consists of three LSTM encoders to capture individual pedestrians’ information, social relationships, and the scene layout. However, it overlooks the weight distribution of neighboring pedestrians, which results in inaccuracies in long-term predictions. The model was evaluated on the ETH, UCY, and Town Center datasets. SR-LSTM [61] is a refined LSTM model that utilizes a message-passing mechanism to iterate over the current state of participants, incorporating the intentions of neighbors. An attention mechanism is applied to assign varying weights to each neighbor. SNS-LSTM [62] enhances the S-LSTM framework by adding navigation and language pooling layers. The navigation pooling mechanism distinguishes equally probable predicted locations, while the language pooling mechanism uses scene segmentation to identify non-crossing areas. This model was evaluated on the ETH and UCY datasets. Song et al. [63] proposed a deep convolutional LSTM network (DCLN) to learn spatio-temporal interactions between pedestrians and their environment. Their approach, which deepens the network and optimizes the number of layers, improves prediction accuracy, even in complex scenarios such as dense evacuations and reverse flows. Salzmann et al. [64] introduced Trajectron++, a modular, graph-based recursive model that predicts trajectories for diverse agents. This model integrates agent dynamics with heterogeneous data, encoding agent interactions, and producing more realistic predictions. The system was evaluated on the ETH, UCY, and nuScenes datasets. Yi et al. introduced FlexiLength-LSTM [65], a single-stream LSTM that automatically adapts to any observation length between 0.3 s and 5 s without retraining. A curriculum-style gate compensates for the distribution shift caused by variable-length inputs, delivering noticeable ADE/FDE improvements over Social-LSTM on ETH/UCY and SDD while keeping the model small and real-time-friendly. Tang et al. proposed HPNet [66], a bidirectional-LSTM encoder–decoder whose hidden states are recursively refined by the model’s own previous predictions through a lightweight historical-prediction attention block. A confidence-based gate decides how much the past prediction should correct the current hidden vector, yielding clearly better long-term FDE on nuScenes-pedestrian and JRDB while maintaining pure RNN complexity suitable for online deployment. Zhang & Liu presented AFC-RNN [67], an adaptive forgetting-controlled RNN that first estimates a memory factor for each historical step via self-attention and then explicitly tunes the forget gate of a standard RNN. The approach noticeably reduces ADE/FDE compared with vanilla LSTM on ETH/UCY, SDD, and NBA datasets, yet still retains a compact recurrent architecture.

Zamboni et al. [68] introduced an innovative 2D convolutional model for pedestrian trajectory prediction based on the CNN architecture. The model encompasses various components, including data pre-processing, trajectory embedding, encoding and decoding, and final prediction. To enhance robustness, they employed data augmentation techniques—such as adding Gaussian noise and performing random rotations, as well as strengthening the model’s representational capacity by increasing the number of channels. Wang et al. [69] proposed a novel model, SEEM, which integrates a generator network and an energy network based on sequence entropy energy. The generator network utilizes an encoder–decoder framework, using historical pedestrian trajectories as input to predict a range of future trajectories. Meanwhile, the energy network ensures the generator produces realistic trajectories by classifying both real and predicted trajectories as true or false. However, the reliance on distance-based features to model human-to-human interactions limits the social acceptability of the trajectory prediction results. To address the potential impact of static obstacles on pedestrian movement, Khel et al. [70] introduced SS-BLSTAS, a spatio-temporal attention and sparse motion field model. This model effectively predicts future pedestrian movements by considering both obstacles and social interactions, utilizing CNN and Bi-LSTM for feature extraction. The model’s performance was evaluated on the ETH and UCY datasets. Yang et al. [71] proposed the IA-LSTM model, which combines spatial feature information of the target pedestrian with the interaction features between pedestrians as inputs to LSTM units for predicting future trajectories. The model incorporates several key modules: the novel Correntropy-based Mechanism, which calculates the Correntropy values between pedestrians and uses them as weights for their interactions, defining personal space through a suitable Correntropy kernel; and the Interaction Module, which extracts the feature representation of interpersonal interactions, dynamically adjusts weights, and calculates the importance of various interactions. Additionally, the model enables the sharing of social information between pedestrians through the interaction module. Despite the significant advancements made by RNN-based prediction models in pedestrian trajectory prediction, they still involve numerous parameters, and the training process often encounters gradient disappearance and explosion issues. Many studies enhance pedestrian interaction by integrating convolutional neural networks, introducing attention mechanisms, and combining scene context to improve prediction accuracy. Table 1 summarizes pedestrian trajectory prediction methods based on RNN.

### 3.2. Method Analysis and Comparison Based on GAN Model

The prediction results of traditional LSTM and Seq2Seq sequence models [72,73] are often based on the average trajectory output. However, pedestrian motion is highly dynamic and random, leading to poor generalization, and the predicted trajectories may not align with physical or social norms. To address these issues and enhance the diversity and rationality of predictions, generative models have been introduced. These models combine sequence models like LSTM with GANs and Variational Autoencoders (VAEs). Recently, many researchers have leveraged the GAN architecture to promote multi-modality in trajectory predictions, as outlined below. Traditional RNN sequence models tend to focus on predicting the “average trajectory”, minimizing the L2 distance between the predicted and actual future trajectory. To overcome the limitations of such models, Gupta et al. [74] proposed the Social-GAN model. This model integrates LSTM sequence prediction with a GAN, where pedestrian motion history is input to the model, and an improved pooling mechanism is introduced. The model also incorporates noise (Z) and uses an enhanced diversity loss function. Compared to models like B-LSTM and S-LSTM, Social-GAN generates more realistic trajectories and significantly improves prediction speed. However, it still relies on a traditional GAN architecture, which leads to unstable training, simplified pedestrian interaction features, and limited generalization capabilities. This model’s performance was evaluated on the ETH and UCY datasets.

Li et al. [75] proposed the Conditional Generation Network (CGNS), which enhances Social-GAN by using static context information and a soft attention mechanism. This model consists of three modules: a deep feature extraction module, a generator module, and a discriminator module, with CNN and GRU serving as feature extractors. Kothari et al. [76] improved on Social-GAN with the introduction of FSGAN, a more advanced GAN architecture. FSGAN integrates two attention modules—physical and social attention—into the model. Additionally, the adversarial loss function of the traditional GAN discriminator has been improved, leading to the development of the S-GAN model with a feedforward discriminator. FSGAN outperforms S-GAN on the ETH and UCY datasets. The Social-BiGAT [77] model is another GAN-based method, but it leverages Graph Attention Networks (GATs). The model includes a generator with an encoder, attention network, and LSTM decoder. Two discriminator modules represent both the current pedestrian and the global scene, while a spatial encoder module encodes scene noise. This architecture enables the generation of multiple trajectories for several individuals in a multi-modal fashion. Amirian et al. [78] introduced the Social-Ways model based on Info-GAN. This model replaces the traditional L2 loss term with an information loss function to capture interactive information. It effectively mitigates mode collapse and dropout issues during GAN training. The model was tested on the ETH and UCY datasets. The Sophie model [79] uses historical trajectories and scene context along with social and physical attention mechanisms to model pedestrian interactions. The model consists of three modules: feature extraction, attention, and a GAN module based on LSTM. The predicted trajectories are constrained by the environment, leading to more accurate and interpretable social and physically feasible outputs. Sun et al. [80] developed a forward–backward prediction network that leverages pedestrian trajectory characteristics. Their reciprocal learning method uses adversarial neural networks to iteratively refine predicted trajectories. The model includes a feature extraction module and a GANs module based on LSTM. Zou et al. [81] proposed the STG-GAN, a scalable spatio-temporal graph adversarial network. This architecture uses a graph structure with attention mechanisms to capture global interactions between pedestrians and scenes. The STG-GAN framework consists of three modules: a feature encoder, a generator/decoder, and a discriminator module. Its performance was evaluated on the ETH and UCY datasets. Most pedestrian trajectory prediction methods rely on 2D image learning. Zhong et al. [82] proposed a 3D depth-PoseGAN method that utilizes a camera system for pose estimation and trajectory prediction in 3D space. This method incorporates Social-GAN for motion prediction, and the PoseGAN framework consists of three components: a human pose estimator, a semantic data generator, and a discriminator. It was tested on a custom dataset. Generative models address the uncertainty in future trajectory predictions by sampling from potential variables. Yang et al. [83] introduced the TPPO model, which consists of three modules: a generator encoding input trajectories, a social attention module for interaction calculation, and a decoder for output generation. The discriminator conducts a min–max game between real and predicted trajectories, and a latent variable predictor estimates the latent variable distribution. This model was tested on the ETH and UCY datasets. Zhou et al. [84] proposed the DACG model, a CVAE-GAN-based model for pedestrian trajectory prediction, incorporating dynamic attention to model time-varying social interactions and pedestrian intentions. The models generator includes a feature encoder, social aggregator, pattern estimator, target estimator, and trajectory decoder. An et al. [85] introduced a gridified map representation for pedestrian state information using a GAN framework. Their model utilizes temporal and spatial attention mechanisms to account for the impact of historical trajectories and spatial interactions. While this model improves stability and accuracy, the introduction of multiple attention mechanisms increases computational complexity. Li et al. [86] proposed POI-GAN, which embeds a “Point-of-Interest” social force layer and a field-of-view mask inside an Info-GAN, enabling the generator to learn trajectories that are attracted to service desks while ignoring occluded neighbors.GANs enhance trajectory prediction not only by improving accuracy but also by ensuring social norms and rationality in the predicted outputs. Many existing GAN architectures are integrated with LSTM networks, and attention mechanisms and Transformer networks are commonly used to enhance interaction modeling and prediction accuracy. Table 2 summarizes various GAN-based pedestrian trajectory prediction methods.

### 3.3. Method Analysis and Comparison Based on GCN Model

Convolutional neural networks (CNNs) [87] are primarily used for computer vision tasks such as classification, detection, tracking, and image segmentation. However, CNNs face challenges in capturing long-term dependencies when processing sequence data, limiting their effectiveness in pedestrian trajectory prediction. Yi et al. [88] were the first to apply CNNs to pedestrian trajectory prediction with their model “Behavior-CNN”. To overcome CNNs’ limitations related to local perception and the lack of long-term dependencies, graph convolutional networks (GNNs) were introduced. GNNs [89] are deep learning models designed specifically for graph-structured data. Unlike traditional neural networks that focus on vector and matrix data, GNNs handle graph data composed of nodes and edges, capturing relationships between them. GNNs have been widely applied in behavior recognition [53], traffic prediction [90], and pedestrian trajectory prediction tasks, demonstrating strong predictive capabilities. By combining GNNs and CNNs, we can use GNNs to learn the relationships and global information among pedestrians while using CNNs to extract spatial features from pedestrian trajectories. This combination enables a more comprehensive modeling of pedestrian trajectory data, leading to more accurate predictions.

Zhang et al. [91] proposed a social graph network for random trajectory prediction, comprising an encoder, a random model, and a decoder. The encoder encodes the graph nodes and edges, learning social interactions and individual representations. The constructed adjacency matrix defines the directed edges between nodes, and the topology of the social graph is dynamically updated based on pedestrian layout and speed. The stochastic model generates latent variables associated with output conditions, while the decoder predicts pedestrian speed and future positions. This model effectively learns social interactions through the social graph network, combining the random model and decoder for trajectory prediction. Mohamed et al. [92] introduced the Social-Spatio-Temporal Graph Convolutional Neural Network (Social-STGCNN), consisting of four components: an input layer, a graph convolutional neural network (GCNN), a temporal convolutional neural network (TCNN), and an output layer. By representing pedestrian trajectories as graphs and using GCNN and TCNN, Social-STGCNN models social relationships between pedestrians, outperforming other models in prediction accuracy, parameter size, and data efficiency. Li et al. [93] expanded on Social-STGCNN by incorporating an attention mechanism, proposing the Attention-GCNN model. This model assigns attention weights to the edges of the graph, aggregating implicit interaction information among pedestrians. Similarly to Social-STGCNN, the AVGCN [94] model combines graph convolutional networks with attention mechanisms to improve prediction accuracy. It estimates the importance of adjacent pedestrians using attention, incorporates learned attention weights into the network, and aggregates the information via GCN. Additionally, it uses a variational trajectory prediction method to account for the randomness in pedestrian movements. Lian [95] proposed the PTP-STGCN model, which selectively models spatial interactions and temporal dependencies among pedestrians. By utilizing a spatio-temporal graph convolutional network and incorporating a T-transformer model, this model better captures social interactions, enhancing prediction accuracy and efficiency. The GraphTCN model [96] is a time-space graph model based on CNNs that uses attention mechanisms and multi-head attention to model spatial and temporal interactions among pedestrians. It also employs an improved temporal convolutional network (TCN) to capture these interactions, while introducing noise in the output layer to generate multiple potential trajectories. Sun et al. [97] proposed the Recursive Social Behavior Graph (RSBG) model, which recursively extracts social relationships to form a social behavior graph. Pedestrians are treated as nodes, and social interactions are propagated through the graph via a graph convolutional network. To enhance trajectory prediction accuracy for multiple road users, Rainbow et al. [98] introduced the Semantics-STGCNN model. This model embeds category information into graph convolutional networks to predict individual trajectories more effectively. A related model, MULTICLASS-SGCN, incorporates multi-class agent embeddings and an adaptive interactive mask to improve training speed and accuracy. Shi et al. [99] proposed the Sparse Graph Convolutional Network (SGCN) to address redundant interactions and overlooked motion trends in pedestrian trajectory prediction. This approach models sparse directed interactions and motion trends, designing an adaptive method to capture interactions and motion trends among pedestrians more effectively. Given that pedestrians often move in groups, the interactions within pedestrian groups can become complex. Zhou et al. [100] proposed the Grouptron model, a dynamic multi-scale prediction framework that incorporates scene-level, group-level, and individual-level graphs to more effectively model the interactions and dynamics among pedestrians. Similar to Grouptron, GroupNet primarily focuses on interaction capture and representation learning. To accurately model asymmetric social interactions among pedestrians, Su et al. [101] introduced a directed graph convolutional network based on prior perception. The network’s design includes three directed graph topologies: view graph, direction graph, and rate graph, which represent the asymmetric social interactions among pedestrians. Bhujel [102] recently introduced a novel method known as DGCN, designed to overcome the limitations in capturing complex interactions among encoded pedestrians, thereby improving the modeling of crowd interactions. This approach decouples spatial and temporal factors by encoding crowd interactions into two low-dimensional latent variable spaces: spatial and temporal latent variables. The author also proposes a new regularization function to capture the true information of each factor. Mi et al. [103] proposed DERGCN, a dynamic-evolving graph convolutional network that online re-wires the interaction graph via learnable edge gates. Instead of using a fixed social adjacency, the model continuously adds/removes edges according to pedestrian motion discrepancy, allowing the GCN to aggregate features only from temporally relevant neighbours. This evolving structure significantly keeps a pure GCN pipeline without external rules. Feng et al. [104] introduced a lightweight CNN-GCN fusion algorithm for on-board deployment. Multi-scale CNN blocks first encode trajectory and scene context; a single GCN layer then fuses trajectory nodes with scene grid-cells, outputting multiple future representations in one forward pass. The hybrid design cuts parameters by 73% versus prior lightweight methods. In recent years, significant progress has been made in prediction tasks through the integration of GCNs, attention mechanisms, and GANs. However, many existing algorithms fail to adequately account for the motion features and deep interactions among agents. The motion patterns and interactions between agents are crucial for accurate predictions, particularly in crowded or highly coordinated scenarios. Future research may focus on designing more sophisticated network architectures and introducing richer attention mechanisms to further enhance the model’s ability to capture complex agent behaviors in intricate scenes. Table 3 summarizes GCN-based pedestrian trajectory prediction methods.

### 3.4. Analysis and Comparison of Methods Based on Transformer Model

The use of Transformer models for pedestrian trajectory prediction has gained considerable attention in recent years. The Transformer, a neural network architecture based on an encoder-decoder structure, self-attention mechanisms, and positional encoding, was originally introduced by [53] for natural language-processing tasks such as machine translation. In the context of pedestrian trajectory prediction, this architecture has been adapted to model the spatial relationships and interactions among pedestrians. These adapted models often incorporate self-attention, multi-head attention, and positional encoding, with variations from the original Transformer model. Notable models such as Social-STGCNN, Trajectron++, and STGAT effectively leverage Transformer principles to model pedestrian interactions. The emergence of Transformer-based pedestrian trajectory prediction methods has advanced this field, achieving significant improvements in prediction accuracy. Recognizing the importance of attention in trajectory prediction, Yu et al. proposed the Space–Time Graph Transformer (STAR) [105] framework. This framework alternately uses spatial and temporal Transformer to capture both spatial and temporal information, while a graph convolution mechanism enables the model to extract complex social interactions. Traditional CNNs or RNNs often require a large number of parameters to model pedestrian movement dynamics and learn long-term temporal dependencies, which results in complex models with low computational efficiency. To address these issues, Yin et al. introduced the Multi-modal Transformer Network (MTN) [106]. This network combines trajectory information with optical flow data, employing attention mechanisms and Transformer modules for feature extraction and fusion, with coarse-grained and fine-grained fusion methods for more accurate trajectory predictions. Conventional sequence modeling methods typically treat time and social dimensions separately, limiting their ability to capture long-term dependencies. To overcome this limitation, Yuan et al. proposed the AgentFormer [107] model, which simultaneously learns representations of both time and social dimensions. This model introduces a novel agent-aware attention mechanism and a multi-agent prediction framework that maintains multi-agent identity information and simulates social influences on other agents’ intentions. This enables effective modeling of social interactions and improved trajectory predictions.

Li et al. [108] developed a graph-based spatial Transformer and a memory playback algorithm to address spatial interactions and temporal consistency. The graph-based spatial Transformer captures pedestrian interactions, while the memory playback algorithm ensures temporal consistency. Additionally, a new evaluation metric, PTU, was proposed to assess the comprehensiveness of multiple future trajectory predictions. Given the uncertainty in pedestrian motion, Gu et al. [109] introduced the MID model, which uses a Transformer architecture to predict random trajectories. The model accounts for motion uncertainty by modeling the prediction problem as an inverse diffusion process within a Markov chain. Liu et al. proposed the Social Graph Transformer Network (SGTN) [110], which integrates graph convolutional networks with Transformer networks to predict trajectories in complex social settings. Unlike previous models, SGTN directly captures spatio-temporal features through an adjacency matrix, rather than structuring the Transformer around the graph framework, improving its ability to predict in complex social environments. Shi et al. [111] introduced the TUTR model, which provides explicit global predictions and implicit pattern-level Transformer parsing of various motion patterns. The model focuses on social interactions through a social-level Transformer decoder and predicts diverse trajectories with associated probabilities through a dual prediction head, eliminating the need for post-processing steps such as clustering. TUTR outperforms methods requiring post-processing in terms of inference speed, particularly in both sparse and dense pedestrian movement scenarios. Despite its strengths, the TUTR model requires extensive parameter tuning, particularly in social force models, and faces challenges in practical applications due to parameter sensitivity. Furthermore, its adaptability to complex data structures, such as map information, remains limited, and its generalization to diverse scenarios requires further investigation. Rei et al. [112] introduced the RNTransformer model, which is divided into two components. First, it utilizes crowd travel information as a new modality to capture global social interactions. The area is divided into nodes, with local models predicting individual pedestrian trajectories within each node, while the global model predicts crowd behavior using sparse spatio-temporal modules. Second, the scene is divided into grids, aggregating pedestrian counts over time to learn crowd behavior, while incorporating a road network model that uses empirical pedestrian trajectories and geometric information to create potential walkable areas. Zhang et al. [113] proposed PPT-Former, a non-autoregressive Transformer trained with Progressive Pretext Tasks. Two learnable prompt embeddings represent unobserved intermediate and destination positions; the model first predicts the endpoint and then generates the whole future sequence in one forward pass. Cross-task knowledge distillation is adopted to avoid catastrophic forgetting between short-term and long-term pretraining stages. Jiang et al. [114] introduced SIAT, a Social Interaction-Aware Transformer that couples a standard Transformer encoder with a dynamic social graph. Nodes are pedestrians and edge strengths are learned from relative positions over time; a lightweight GCN extracts spatial features while the Transformer captures temporal dynamics. A single Transformer decoder fuses both modalities, enabling the model to reason about collective motion without hand-crafted social rules and delivering clear error reductions in dense scenes. Lian et al. [115] presented ASTRA, a scene-aware Transformer designed for edge deployment. A U-Net backbone extracts bird’s-eye-view scene context; a graph-aware Transformer encoder models social interactions, and a CVAE-based decoder produces deterministic or stochastic predictions. A weighted penalty loss further enforces scene compliance. The entire pipeline integrates scene, spatial, temporal, and social cues within a lightweight Transformer stack, achieving seven-fold parameter reduction. Pedestrian trajectory prediction based on Transformer models presents several challenges. While the Transformer excels in sequence modeling, its effectiveness can be limited by data quality and model design. Therefore, future work should consider improving these models by incorporating attention mechanisms, combining them with other architectures, and leveraging multi-scale features. Overall, pedestrian trajectory prediction using Transformer models is a complex task that requires careful data preprocessing, feature extraction, and optimization of model training. Continuous research and experimentation will lead to improvements in accuracy and robustness. Table 4 summarizes some Transformer methods for pedestrian trajectory prediction.

## 4. The Analysis of Multi-Modal Pedestrian Trajectory

The movement behavior of pedestrians is multi-modal and uncertain. Based on the historical trajectories of pedestrians and the surrounding environmental information, there can be multiple plausible future trajectories for each pedestrian. This concept of predicting multiple reasonable trajectories for agents is referred to as multi-modal trajectory prediction (MTP), which aims to generate a diverse, acceptable, and interpretable distribution of future predictions for each agent. Trajectory prediction tasks are typically categorized into two types: deterministic trajectory prediction (DTP) and multi-modal trajectory prediction (MTP). DTP provides a single prediction result for each agent, where the model predicts one trajectory based on historical data, without considering uncertainties or alternative outcomes. This type of prediction is useful in simpler, more predictable environments. However, due to the numerous unknown factors involved in pedestrian behavior, it is unrealistic for DTP to predict a single trajectory without adequate information. In contrast, multi-modal trajectory prediction generates multiple, diverse, socially acceptable predictions for each pedestrian. MTP accounts for the inherent uncertainty and variability in pedestrian movement, providing a richer and more flexible prediction. This approach reflects the reality that pedestrians may take different paths based on varying circumstances, such as interactions with other pedestrians or the environment. MTP is more suitable for complex, dynamic scenarios where a single trajectory prediction may not be sufficient. The DTP framework typically follows a sequence-to-sequence structure, where the encoder extracts spatial and temporal information from historical data, while the decoder is responsible for predicting the future path based on the encoded information. In comparison, MTP models aim to generate a diverse, socially acceptable, and interpretable distribution of future trajectories. While DTP provides a single trajectory prediction, MTP faces challenges in ensuring that the generated predictions are both realistic and reflective of the diverse possibilities in human behavior. Many advanced frameworks have been proposed to tackle these challenges, some of which are compared in Table 5.

## 5. Summary of Pedestrian Trajectory Prediction Methods

Over the past decade, pedestrian trajectory prediction has evolved from simple linear models to sophisticated deep learning frameworks capable of capturing complex social dynamics and multi-modal futures. Early approaches primarily relied on hand-crafted rules or Gaussian processes, but the advent of deep neural networks has enabled data-driven modeling of both individual motion patterns and group interactions. RNNs excel at capturing sequential dependencies in trajectory data due to their inherent temporal modeling bias, enabling effective learning of movement patterns across consecutive time steps. However, their sequential processing suffers from vanishing gradients over long sequences and inherently non-parallelizable computation, limiting both training efficiency and real-time inference, with recent work increasingly adopting parallelizable alternatives for long-horizon prediction. GANs represent a paradigm shift toward probabilistic forecasting, enabling the generation of multiple socially plausible trajectories rather than single-point estimates to explicitly address the stochastic nature of human navigation in shared spaces. Key challenges include well-documented issues such as training instability and mode collapse inherent to adversarial learning, while the stochasticity of outputs complicates verification in safety-critical applications requiring deterministic guarantees, now increasingly mitigated by diffusion-based uncertainty quantification. GNNs provide a natural abstraction for modeling pedestrian interactions by representing social scenes as dynamic graphs, thereby enabling explicit reasoning about relational constraints and spatial influences.

However, computational complexity scales non-linearly with agent density, creating bottlenecks in crowded scenarios, and standard GNNs typically require integration with dedicated temporal modules to capture full spatio-temporal dynamics, with recent advances in optimizing them via lightweight scene-aware architectures. Transformer architectures have redefined trajectory prediction through self-attention mechanisms that model global context across entire observation sequences—particularly advantageous for long-range dependencies in complex multi-agent settings. Their parallelizable design offers significant training efficiency gains, though self-attention’s permutation-invariance requires explicit positional encoding to preserve critical temporal structure, and the large parameter count of typical models demands substantial computational resources and extensive datasets, prompting current research toward efficient variants that reduce parameter counts while maintaining global context awareness. This systematic analysis reveals complementary strengths and limitations across architectural paradigms, with Korbmacher and Tordeux [121] noting that while deep learning has surpassed traditional knowledge-based methods in local trajectory prediction accuracy, its capability for large-scale simulation and collective dynamics remains to be validated, suggesting that hybrid approaches combining both methodologies may offer the most promising path forward, now converging toward integrated frameworks combining relational reasoning (GNNs), global contextual modeling (Transformer), and calibrated generation (diffusion models) with explicit emphasis on computational efficiency and verifiability for real-world deployment.

## 6. Datasets and Performance Evaluation

### 6.1. Dataset

In pedestrian trajectory prediction, dataset selection is as crucial as model choice and parameter tuning. A high-quality dataset not only enables the model to better capture complex trajectory patterns but also enhances the accuracy and robustness of predictions in real-world scenarios. An appropriate dataset provides comprehensive validation across diverse settings, forming a strong foundation for reliable prediction outcomes. The ETH [122] and UCY [123] datasets are among the most commonly used in this field. These datasets provide marked position coordinates of pedestrians based on surveillance videos, covering five scenarios. The ETH dataset includes the ETH and Hotel datasets, while the UCY dataset consists of three: Univ, Zara1, and Zara2. The ETH dataset features pedestrian trajectory data in complex environments such as shopping malls, railway stations, and airports, offering high-quality video and precise pedestrian location labeling. The UCY dataset captures pedestrian movement in various settings, including campuses, streets, and squares. A common approach for training and testing on this dataset is the leave-one-out method. In addition to ETH and UCY, several other notable datasets include the Stanford Drone Dataset [124], which captures interactions between pedestrians, bicyclists, and cars using drone footage from a campus environment, providing pixel coordinates for each agent’s trajectory over approximately 5 h of footage; the JAAD dataset [125], which focuses on pedestrian behavior at street crossings, offering detailed behavior labels of pedestrians over approximately 240 h of video footage; the nuScenes dataset [126], which captures complex urban environments involving vehicles, pedestrians, and cyclists in urban streets and intersections, and includes sensor data from RADAR and LIDAR, covering 1000 scenes. Additional datasets include the Argoverse dataset [127], which contains 2D and 3D tracking data for vehicles, pedestrians, and cyclists, with detailed map information for urban streets and intersections, covering around 320 h of interaction data; and the PIE dataset [128], which focuses on pedestrian behavior at crosswalks and sidewalks, including trajectories, gaze data, and behavior annotations for around 6 h of recordings. These datasets offer a rich source of information for understanding pedestrian movement and interactions, facilitating the development of models that can capture the complexities of human behavior in a wide range of environments, from controlled campuses to dynamic urban intersections. Table 6 provides detailed information about the ETH-UCY dataset, while Table 7 offers a summary of commonly used pedestrian trajectory datasets.

### 6.2. Evaluation Indicators

The evaluation of pedestrian trajectory prediction models requires a variety of metrics to assess performance from different perspectives. These metrics are typically categorized into Essential Evaluation Metrics, Advanced Trajectory Metrics, and Probabilistic Distribution Metrics. Each category provides valuable insights into specific aspects of model performance, such as accuracy, precision, and the ability to predict multiple plausible future trajectories. Essential Evaluation Metrics are commonly used to measure the basic accuracy of predictions. These include Accuracy, Precision, Recall, F1 Score, Mean Absolute Error (MAE), and Root Mean Squared Error (RMSE). Accuracy refers to the proportion of correctly predicted trajectory points relative to the total number of points. Precision and Recall evaluate the accuracy of predicted positive instances, with Precision focusing on the proportion of true positives among all predicted positives, and Recall assessing the proportion of true positives among all actual positives. The F1 Score, the harmonic mean of Precision and Recall, provides a balanced measure of model performance. MAE calculates the average absolute difference between predicted and actual trajectory points, while RMSE emphasizes larger errors by computing the square root of the average squared differences between predicted and true values. While these essential metrics provide a solid foundation for basic performance evaluation, Advanced Trajectory Metrics are needed to capture more nuanced spatial and temporal precision in trajectory predictions. Kothari et al. [129] introduced an interaction-centric benchmark (TrajNet++) with collision-based evaluation metrics, demonstrating significant performance gains of interaction-aware models over non-interaction models in crowded environments.

Moreover, Average Displacement Error (ADE) and Final Displacement Error (FDE) are key metrics for understanding the accuracy of both the overall trajectory and its final position. ADE measures the average Euclidean distance between predicted and true trajectories across all time steps, offering a comprehensive view of the models accuracy over time. In contrast, FDE specifically measures the distance between the final predicted position and the true final position, providing a direct assessment of endpoint prediction accuracy. Additionally, Minimum Average Displacement Error (minADE) and Minimum Final Displacement Error (minFDE) focus on identifying the model with the least errors among multiple predicted trajectories, which is crucial when precise final position or long-term trajectory accuracy is required. Probabilistic Distribution Metrics, such as Negative Log-Likelihood (NLL) and Kernel Density Estimation NLL (KDE-NLL), offer deeper insights into the model’s uncertainty and prediction quality. NLL evaluates how well the model’s probability distribution aligns with the true trajectory, with a lower NLL indicating a better fit. KDE-NLL extends this concept by using kernel density estimation to capture the multi-modal nature of trajectory distributions, making it especially valuable for tasks involving the prediction of multiple potential outcomes or significant uncertainty. These probabilistic metrics are essential for models that generate probability distributions, particularly in multi-modal prediction tasks, where understanding the spread and confidence of predictions is critical. By integrating these evaluation metrics, a comprehensive assessment of model performance can be achieved. This enables more targeted improvements and optimizations in pedestrian trajectory prediction tasks. Collectively, these metrics provide a deeper understanding of model performance, particularly in terms of uncertainty and the distribution of future trajectories. While NLL focuses on pointwise likelihood, KDE-NLL refines this by considering the overall distribution fit, offering a more holistic view of prediction quality. Table 8 presents a classification of these evaluation metrics.

### 6.3. Performance Comparison

**Figure 5:**
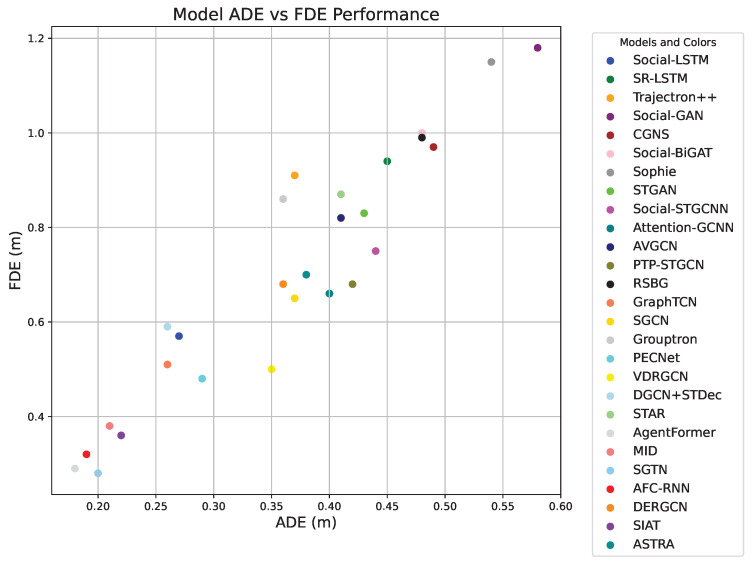
The AVG Of ADE/FDE comparison on dataset.

**Figure 6:**
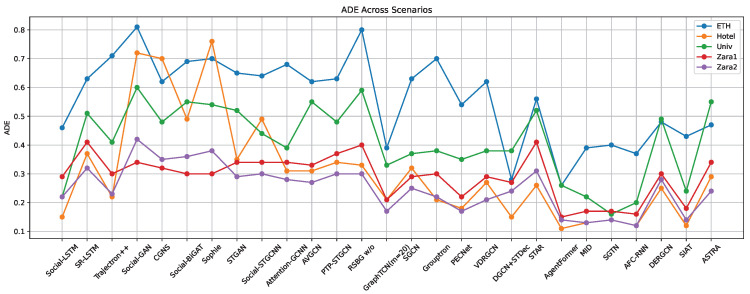
The ADE values of different models are compared in the ETH-UCY dataset.

**Figure 7:**
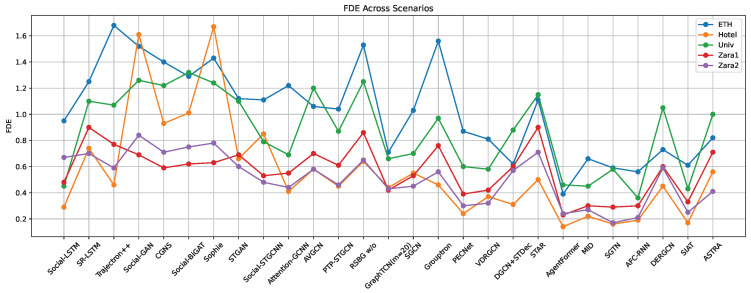
The FDE values of different models are compared in the ETH-UCY dataset.

Currently, the ETH-UCY dataset is widely used in pedestrian trajectory prediction. To better compare the performance of each model, this paper focuses solely on evaluating the models using this dataset. Refer to Table 9 and Figure 5, Figure 6 and Figure 7 for the comparison results. According to Table 9 and Figure 5, Figure 6 and Figure 7, the prediction accuracy of models based on RNNs is generally lower compared to those based on GANs, GCNs, and Transformer-based models across different scenarios in the ETH-UCY dataset. The trends observed in the graphs indicate that GANs-based methods are capable of generating multiple plausible trajectory models, which outperform models that predict only a single trajectory. Additionally, many models improve prediction accuracy by integrating various techniques. For example, models like FSGAN and Sophie incorporate attention mechanisms, while others, such as Social-BiGAT, Social-STGCNN, AVGCN, and GraphTCN, leverage graph neural networks. The prediction accuracy of Social-BiGAT and Social-STGCNN is approximately 50% higher than that of S-LSTM, with the ADE and FDE of GraphTCN reaching 0.26 and 0.51, respectively. The STAR model, which uses the Transformer models for long-term dependency modeling, achieves superior accuracy compared to RNN-based methods. Transformer-based models, such as AgentFormer, MID, and SGTN, incorporate various attention mechanisms and combine graph neural networks with other techniques, resulting in better prediction accuracy than their counterparts. In conclusion, with the ongoing development of deep learning technologies, integrating the strengths of different models such as incorporating attention mechanisms, leveraging scene semantics, and integrating multi-modal features can significantly enhance the accuracy of pedestrian trajectory predictions [130,131].

## 7. Rethinking Future Directions

Integrating our comprehensive analysis, we identify that the field’s core challenge is not technical precision enhancement, but rather theoretical poverty at the cognitive level: current deep learning models lack integration of fundamental theories about human mobility behavior. Social psychological research demonstrates that human trajectory decisions are governed by three principles: the efficiency principle (minimizing effort), the safety principle (avoiding risk), and the social norm principle (following implicit rules), yet current methods explicitly model only the first. This cognition–technology gap results in the ‘high-accuracy, low-acceptance paradox’: models perform excellently on numerical metrics but generate socially counterintuitive trajectories in real scenarios. Bridging this gap requires a new paradigm of ‘theory-guided data-driven’ approaches: integrating mature theories from social psychology (such as the Theory of Planned Behavior and social force models) as inductive biases into deep architectures, rather than relying solely on pure data-driven learning. We predict breakthroughs will emerge from three integration directions: architectural fusion of cognitive theory and deep learning, temporal fusion of multi-modal perception and behavior prediction, and scale fusion of individual decision-making and crowd dynamics. This paradigm shift will elevate pedestrian trajectory prediction from an ‘engineering optimization problem’ to a ‘human behavior understanding tool’, providing autonomous systems with genuine social intelligence.

### 7.1. Semantic Scene Comprehension

Advanced pedestrian trajectory prediction increasingly leverages rich contextual data to significantly enhance accuracy. However, to transform this broad concept into actionable research, future work should focus on developing concrete methods for semantic scene comprehension. For instance, UAV-based models [132] with top-view perspectives have demonstrated advantages in capturing comprehensive traffic patterns in complex environments like multilane roundabouts. One promising approach is to integrate computer vision techniques with sensor fusion, using convolutional neural networks (CNNs) to extract detailed scene information (e.g., road layouts, building structures, pedestrian zones) from high-resolution images or video streams. This visual data can be combined with information from other sensors such as LiDAR, radar, and GPS to construct a comprehensive spatial representation of urban environments. Moreover, natural language processing (NLP) techniques can be applied to metadata or contextual descriptions (e.g., urban planning documents) to enhance scene understanding further. A technical roadmap could involve the following: (1) developing a multi-modal data fusion framework that integrates image, LiDAR, and contextual text data; (2) designing specialized CNN architectures for robust feature extraction under varied lighting and weather conditions; (3) implementing GNNs to model the relationships between different scene elements and pedestrian behaviors. This detailed approach will bridge the gap between abstract semantic understanding and practical trajectory prediction.

### 7.2. Robust Integration and Transferability

The deployment of pedestrian trajectory prediction systems across diverse scenarios necessitates robust integration and transferability. To achieve this, research should focus on developing models that not only perform well in a single environment but also adapt to new contexts through domain adaptation and transfer learning techniques. In this context, continual learning models [133] offer promising solutions to overcome catastrophic forgetting when models face dynamically changing environments. For instance, an effective strategy may involve pretraining models on large-scale datasets from one urban area and then fine-tuning them on smaller datasets from new cities using techniques such as adversarial domain adaptation. Additionally, multi-task learning frameworks that jointly optimize for trajectory prediction and related tasks (e.g., traffic flow estimation) can facilitate knowledge transfer across domains. A concrete technical roadmap might include the following: (1) creating benchmark datasets that represent a variety of urban settings with different cultural and infrastructural characteristics; (2) exploring adversarial training schemes to minimize domain discrepancies; (3) implementing online learning mechanisms that continuously update the model as new data is acquired. These steps will enhance the adaptability of trajectory prediction systems and ensure seamless integration with existing urban infrastructure.

### 7.3. Creating Comprehensive Benchmarks and Datasets

Progress in pedestrian trajectory prediction hinges on the availability of comprehensive, standardized benchmarks and datasets. To move beyond general recommendations, future research should prioritize the creation of large-scale, diverse datasets that capture a wide range of urban scenarios, including complex interactions, heterogeneous agents, and environments with nonlinear constraints. This can be achieved by leveraging both real-world data collection (e.g., through partnerships with municipal authorities and smart city projects) and simulation environments that mimic diverse urban dynamics. Furthermore, establishing public benchmarks with standardized evaluation protocols and pretrained models will lower the barrier to entry for new researchers and promote reproducibility. A technical roadmap in this area could involve the following: (1) designing data collection campaigns that cover different times of day, weather conditions, and urban events; (2) developing synthetic data generators that can simulate rare or dangerous scenarios; (3) creating an open-source platform for dataset sharing and benchmark evaluation. Such initiatives will drive the field forward by providing the necessary resources for rigorous model testing and development.

### 7.4. Adaptive Prediction with Deep Learning and Reinforcement Learning

Deep learning and reinforcement learning have shown great promise in advancing pedestrian trajectory prediction, yet their full potential remains underexplored in integrated frameworks. Future research should aim to develop adaptive models that combine the strengths of both approaches. For example, deep reinforcement learning (DRL) can be used to learn adaptive decision-making policies that adjust to real-time changes in the environment, while deep neural networks (e.g., RNNs, CNNs, GNNs) can extract and fuse high-dimensional features from diverse data sources.A concrete technical roadmap could involve (1) constructing a DRL-based framework where agents learn optimal prediction strategies through interaction with a simulated environment, (2) incorporating attention mechanisms within GNNs to dynamically model social interactions, and (3) integrating multi-modal sensor data using advanced fusion algorithms. This hybrid approach will allow for continuous learning and refinement, ultimately leading to more nuanced and accurate trajectory predictions in complex, real-world scenarios.

### 7.5. Balancing Precision and Efficiency

As predictive models become increasingly complex, balancing accuracy with computational efficiency is essential for real-time deployment. Future models must be designed to deliver high precision while operating within the constraints of available computational resources. This requires not only algorithmic improvements but also innovations in model architecture and hardware utilization. Researchers should explore techniques such as model compression, pruning, and knowledge distillation to reduce model size and inference time without significantly compromising accuracy. Moreover, implementing parallel and distributed computing frameworks, as well as leveraging hardware accelerators like GPUs and TPUs, can substantially improve performance. A detailed technical roadmap might include (1) benchmarking existing models to identify computational bottlenecks, (2) developing lightweight neural network architectures specifically tailored for real-time inference, and (3) deploying these models on edge devices to assess their performance in practical, large-scale urban applications. Balancing precision and efficiency in this manner will be crucial for the practical adoption of pedestrian trajectory prediction systems in smart city infrastructures.

## 8. Conclusions

This survey has provided a comprehensive examination of deep learning approaches for pedestrian trajectory prediction, highlighting the evolution from basic RNN architectures to sophisticated Transformer and diffusion models. Through systematic categorization and critical analysis of RNNs, GANs, GCNs, and Transformer, we have identified the unique advantages and inherent limitations of each paradigm. Our comparative framework, presented in structured tables, enables direct assessment of model capabilities across multiple dimensions, including social interaction modeling, multi-modal output generation, and long-term dependency capture. The review contributes three key aspects to the field: a systematic classification of evaluation methodologies that fills gaps in the existing literature, a critical synthesis of methodological evolution in Section 5 that traces how successive approaches address prior limitations, and future directions tightly coupled with identified research gaps. Specifically, we have outlined concrete research paths addressing semantic scene comprehension, robust cross-domain transfer, and computational efficiency challenges. These contributions advance the development of more reliable, socially compliant, and computationally efficient trajectory prediction systems, with significant implications for autonomous driving, robot navigation, and intelligent surveillance. By providing both a historical perspective and forward-looking analysis, this survey serves as a valuable resource for researchers and practitioners working to enhance pedestrian safety and mobility in complex urban environments.

## Figures and Tables

**Table 1:** Critical comparison of methods based on Recurrent Neural Networks.

Method	Ref.	Contribution	Strengths	Limitations
S-LSTM	[14]	Introduced social pooling to share hidden states among pedestrians.	Social pooling	Local interaction, ignoring contextual information.
ST-RNN	[54]	Used a transfer matrix to capture local spatio-temporal contexts.	Models time-specific and location-specific transitions.	Predefined tensor structures lack flexibility.
DESIRE	[55]	An encoder–decoder framework with a VAE for diverse trajectory generation.	Inverse optimal control and long-term prediction.	Spatial constraints.
S-aware LSTM	[56]	Embedded prior knowledge via a recursive Gaussian process for crowd dynamics.	Suitable for unstructured scenarios.	Weak generalization.
MX-LSTM	[57]	Integrated head orientation and location information into LSTM.	Long-term prediction, joint prediction.	Limited application scenarios.
S-Attention	[58]	Used a spatio-temporal graph with soft attention to model pedestrian influences.	Social interaction, attention mechanism.	Maintained the entire image.
S-Scene-LSTM	[60]	A hierarchical LSTM combining individual, social, and scene layout information.	Scene layout.	Short trajectory prediction is not accurate.
SR-LSTM	[61]	Refined pedestrian states via a message-passing mechanism and attention.	Joint prediction.	No consideration of static scene context information.
SNS-LSTM	[62]	Extended S-LSTM with navigation and language pooling layers.	Multi-mode input.	Complex interaction.
DCLN	[63]	A deep convolutional LSTM network for learning spatio-temporal interactions.	Wide coverage.	The model is complex.
Trajectron++	[64]	A modular, graph-based model integrating agent dynamics and heterogeneous data.	Dynamic constraints, scene information, probability distribution.	Weak generalization.
2D CNN	[68]	Applied a 2D CNN architecture for trajectory prediction with data augmentation.	Simple yet effective; benefits from standard CNN augmentation techniques.	Lacks inherent sequential modeling capability.
SEEM	[69]	A generator-energy network based on sequence entropy energy.	Probability distribution.	Insufficient consideration of scene semantic information.
SS-BLSTAS	[70]	A spatio-temporal model considering obstacles and social interactions.	CNN combined with attention, comprehensive interaction.	Poor generalization ability.
IA-LSTM	[71]	Combined spatial and interaction features using a Correntropy-based mechanism.	Enables information sharing among pedestrians and captures complex interactions in crowds.	Mediocre performance in diverse scenarios and emergency situations.
FlexiLength-LSTM	[65]	Length-adaptive LSTM with curriculum gate to counter distribution shift under variable observation lengths.	Single model for any length; tiny param. count; real-time; clear ADE/FDE boost.	Uni-directional; long-horizon drift; no explicit social pooling.
HPNet	[66]	Bi-LSTM whose hidden states are refined by its own past predictions via historical-prediction attention and confidence gate.	Pure RNN; better long-term FDE; online capable; plug-and-play.	Extra cache/compute; rudimentary social modeling.
AFC-RNN	[67]	Adaptive forgetting-controlled RNN that uses self-attention memory factors to tune the forget gate explicitly.	Compact; keeps recurrent pipeline.	Still uni-directional; limited scene context.

**Table 2:** Critical comparison of GANs-based pedestrian trajectory prediction methods.

Method	Ref.	Contribution	Strengths	Limitations
Social-GAN	[74]	Combines LSTM with GANs for realistic trajectory generation, but suffers from unstable training and limited generalization.	Multi-modal output	Limited interaction modeling, unstable training.
CGNS	[75]	Enhances Social-GAN by adding static context and attention mechanism, improving feature extraction.	Probability distribution, joint optimization	Complex structure.
FSGAN	[76]	Improved GANs with physical and social attention, outperforming Social-GANs in some datasets.	Diverse trajectory generation	Weak social interaction modeling.
S-BiGAT	[77]	Uses graphical attention (GAT) with a generator and LSTM decoder to produce multi-modal predictions.	Multi-modal output	Strong feature dependency.
Social-Ways	[78]	Replaces L2 loss with information loss, reducing mode collapse and improving training stability.	Improved generalization	Limited applicability.
Sophie	[79]	Uses attention mechanisms and GANs with LSTM to provide more interpretable and accurate trajectory predictions.	Incorporates scene semantics	Requires manual feature sorting.
Forward-Backward	[80]	Uses reciprocal learning and adversarial networks to refine predictions.	Reciprocal constraints	Slow fitting.
STG-GAN	[81]	A scalable spatio-temporal GAN, using attention for global interactions.	Scalable interaction modeling	Misconnection between images.
Depth-PoseGAN	[82]	A 3D pose and trajectory prediction method based on GANs for motion prediction in 3D space	Effective in 3D prediction	High computational complexity.
TPPO	[83]	Uses GANs with social attention and a min–max game for trajectory refinement.	Latent variable exploration	Lacks robustness in interaction modeling.
DACG	[84]	A CVAE-GAN model with dynamic attention to model interactions and intentions.	Dynamic encoding, multi-modal output	Complex model structure.
SGMA-GAN	[85]	Uses GANs with temporal and spatial attention for dynamic interaction modeling	State stability, dynamic interaction	High computational complexity.
POI-GAN	[86]	Info-GAN with POI social force layer and field-of-view mask to model service-scene attraction.	Handles POI attraction; FOV filters unrealistic links; faster convergence.	Extra POI labels needed; still frame-based social pool.

**Table 3:** Critical comparison of methods based on Graph Convolutional Neural Networks.

Method	Ref.	Contribution	Strengths	Limitations
Behavior-CNN	[88]	First CNN model applied to pedestrian trajectory prediction, establishing the viability of convolutional approaches for this task.	Pioneering CNN application	Local receptive fields; no long-term dependencies
Social Graph Network	[91]	Encoder–decoder framework with dynamic adjacency matrix that learns evolving social interactions through graph structures.	Dynamic social modeling	Computationally intensive; data-dependent
Social-STGCNN	[92]	Integrates graph convolutional networks with temporal convolutional networks to jointly model social relationships and spatio-temporal dependencies.	Compact architecture; fast inference	Computationally demanding; low interpretability
Attention-GCNN	[93]	Enhances Social-STGCNN with attention mechanisms to dynamically weight the importance of different pedestrian interactions.	Selective attention; improved accuracy	Black-box nature
AVGCN	[94]	Combines attention mechanisms with variational trajectory prediction, incorporating visual field constraints for more realistic interaction modeling.	Field-of-view constraints; uncertainty handling	Poor crowd performance; data-sensitive
PTP-STGCN	[95]	Employs spatio-temporal graph convolutional networks with T-transformer model to capture both spatial interactions and temporal dependencies.	Flexible architecture	High complexity
GraphTCN	[96]	Unified time-space graph model utilizing CNNs with multi-head attention to capture interactions and generate diverse potential trajectories.	Efficient reasoning; adaptive gating	Limited spatial modeling
RSBG	[97]	Recursively extracts social relationships to construct dynamic social behavior graphs that evolve over time.	Social norm modeling	Parameter-sensitive
Semantics-STGCNN	[98]	Incorporates semantic category information into graph convolutional networks to enable trajectory prediction for diverse road users.	Multi-class prediction	High computation; limited cross-category interactions
SGCN	[99]	Focuses on sparse-directed interactions and motion trend modeling to reduce redundant computations in crowded scenes.	Sparse interaction efficiency	Subjective interactions
Grouptron	[100]	Multi-scale hierarchical framework incorporating scene-level, group-level, and individual-level graphs for comprehensive pedestrian dynamics modeling.	Multi-scale representation	Low interpretability
Directed Graph CNN	[101]	Models asymmetric social interactions using directed graph topologies based on visual perspective, movement direction, and velocity relationships.	Asymmetric social modeling	Limited scenario validation
DGCN	[102]	Decouples spatial and temporal factors into separate latent spaces with specialized regularization for improved crowd interaction modeling.	Disentangled learning	Data- and computation-intensive
DERGCN	[103]	Dynamic-evolving GCN: learnable edge gates add/remove neighbors online, keeping only temporally relevant connections.	Pure GCN; no hand-crafted rules.	Edge-gate training needs careful regularization; density hyper-param-sensitive.
CNN-GCN Fusion	[104]	Lightweight multi-scale CNN and single GCN layer fuses trajectory nodes with scene grid-cells; one-shot forward.	Params decling	Scene-grid resolution sensitive; single GCN may under-fit dense crowds.

**Table 4:** Critical comparison of methods based on Transformer Neural Networks.

Method	Ref.	Contribution	Strengths	Limitations
STAR	[105]	Alternates between spatial and temporal Transformer to capture both spatial and temporal information, using graph convolution to model social interactions.	Space–time interaction, self-attention	High information dependency, complex computation.
MTN	[106]	Combines trajectory and optical flow data, using attention mechanisms and Transformer modules for feature extraction and fusion for accurate predictions.	Multi-mode input, fine-grained representation	Strong data dependency, complex training.
Agent- Former	[107]	Simultaneously learns time and social dimensions, using an agent-aware attention mechanism and a multi-agent framework to model social interactions and improve predictions.	Models temporal and social aspects, preserves identity info	High data dependency, high computation.
GS Transformer	[108]	Uses a spatial Transformer to capture pedestrian interactions and a memory playback algorithm to ensure temporal consistency in trajectory predictions.	Strong spatial interaction, smooth trajectories	High computational complexity.
MID	[109]	Predicts random trajectories using a Transformer architecture, modeling motion uncertainty as an inverse diffusion process within a Markov chain.	Combines diffusion and reverse processes	Low feasibility.
SGTN	[110]	Integrates graph convolutional networks and Transformer networks, directly capturing spatio-temporal features through adjacency matrices for complex social environment predictions.	Handles complex interactions, long-term dependencies	Complex calculations, difficult parameter tuning.
TUTR	[111]	Provides global predictions and pattern-level parsing using a dual prediction head and social-level Transformer decoder, eliminating post-processing steps like clustering.	Fast, efficient prediction	Needs better adaptability to complex data.
RN- Transformer	[112]	Uses crowd travel information for global social interaction modeling; divides the area into nodes and grids for local and global trajectory prediction, integrating a road network model.	Captures global social interactions, explores road networks	Complex optimization, many hyperparameters.
PPT-Former	[113]	Non-autoregressive Transformer that uses learnable destination prompts and progressive pretext tasks to generate full trajectories in one shot, with cross-task distillation for smoother long-term motion.	Single-shot generation; cross-task distillation; smoother paths; noticeable error drop.	Two-stage training overhead; prompt length sensitive; no explicit scene context.
SIAT	[114]	Social Interaction-Aware Transformer that learns time-varying social graph edge weights from relative motion and fuses parallel GCN spatial interactions with Transformer temporal dynamics inside a single decoder.	Captures both temporal and social without hand-crafted rules; clear accuracy gain in crowds	Graph density hyper-param; GCNs branch adds memory; scene raster unused.
ASTRA	[115]	Lightweight scene-aware Transformer that uses a U-Net backbone to extract bird’s-eye scene context once in a graph-aware encoder, produces deterministic or stochastic trajectories via a CVAE decoder, and penalises non-walkable paths with a weighted scene-compliance loss.	Params decling; stochastic outputs	U-Net scene needs calibration; CVAE latent variance tricky; penalty weight tuned per dataset.

**Table 5:** Comparison of pedestrian trajectory prediction methods.

Method	References	Description, Advantages, and Shortcomings
Noise-based prediction	[55,74,76,78,107,116]	Adds random noise to deterministic predictions, generating multiple outcomes. Simple but lacks accuracy and increases complexity.
Anchor-based prediction	[117,118,119]	Uses anchor points for trajectory prediction, improving robustness but difficult anchor selection.
Grid-based prediction	[108,120]	Represents locations with grid maps for predictions. Flexible but computationally expensive and sensitive to grid resolution.
Bivariate Gaussian output	[92]	Assumes bivariate Gaussian distribution for positions, allowing multi-modal predictions but unrealistic and computationally costly.

**Table 6:** ETH-UCY Datasets.

Dataset	View	Scene	Frame	Number of People	Size of Group	Number of Obstacles
ETH	Bird View	ETH	1448	360	243	44
		Hotel	1168	390	326	25
UCY	Bird View	Univ	541	434	294	16
		Zara1	866	148	91	34
		Zara2	1052	204	140	34

**Table 7:** Summary of pedestrian trajectory prediction datasets.

Dataset	Year	Agents	Scene	Durations	Data Format
UCY	2007	Pedestrian	Campus, Urban	29.5 min	Frame sequences with pedestrian coordinates
ETH	2009	Pedestrian	Campus, Sidewalks, Hotel	25 min	Timestamps and 2D coordinates on a plane
Stanford Drone	2016	Pedestrian, Bicyclists, Cars	Campus	5 h	Pixel coordinates for each agent’s trajectory
JAAD	2017	Pedestrian	Crossing streets	240 h	Video frames with pedestrian behavior labels
Nuscenes	2019	Vehicles, Pedestrians, Cyclists	Urban streets, intersections	1000 scenes	Sensor data including RADAR and LIDAR
Argoverse	2019	Vehicles, Pedestrians, Cyclists	City streets, intersections	320 h	2D and 3D tracking data with detailed map info
PIE	2019	Pedestrian	Crosswalks, sidewalks	6 h	Trajectories, gaze data, behavior annotations

**Table 8:** ADE/FDE comparison of each method on the ETH-UCY dataset.

Class	Evaluation Metrics	Formula and Description
Essential Evaluation Metrics	Accuracy	$\mathrm{Accuracy}=\frac{N_{\mathrm{correct}}}{N_{\mathrm{total}}}$, where $N_{\mathrm{correct}}$ = Number of correct predictions, $N_{\mathrm{total}}$ = Total number of predictions.
	Precision	$\mathrm{Precision}=\frac{TP}{TP+FP}$, where TP = True Positives, FP = False Positives.
	Recall	$\mathrm{Recall}=\frac{TP}{TP+FN}$, where TP = True Positives, FN = False Negatives
	F1 Score	$F1=2\times \frac{\mathrm{Precision}\times \mathrm{Recall}}{\mathrm{Precision}+\mathrm{Recall}}$, the harmonic mean of Precision and Recall, providing a balance between the two metrics.
	Mean Absolute Error (MAE)	$\mathrm{MAE}=\frac{1}{n}\sum_{i=1}^{n}\left|{\hat{y}}_{i}-y_{i}\right|$, where ${\hat{y}}_{i}$ is the predicted value for the *i*-th data point, $y_{i}$ is the actual ground-truth value for the *i*-th data point.
	Root Mean Squared Error (RMSE)	$\mathrm{RMSE}=\sqrt{\frac{1}{n}\sum_{i=1}^{n}{\left({\hat{y}}_{i}-y_{i}\right)}^{2}}$.
Advanced Trajectory Metrics	Average Displacement Error (ADE)	$\mathrm{ADE}=\frac{\sum_{i=1}^{N}\sum_{t=t_{obs}+1}^{t_{pred}}{∥Y_{i}^{t}-{\hat{Y}}_{i}^{t}∥}_{2}}{N\times (t_{pred}-t_{obs}-1)}$, where $∥Y_{i}^{t}-{\hat{Y}}_{i}^{t}{∥}_{2}$ is the Euclidean distance between $Y_{i}^{t}$, the actual position of pedestrian *i* at time *t*, and ${\hat{Y}}_{i}^{t}$, the predicted position.
	Final Displacement Error (FDE)	$\mathrm{FDE}=\frac{\sum_{i=1}^{N}{∥Y_{i}^{t_{pred}}-{\hat{Y}}_{i}^{t_{pred}}∥}_{2}}{N}$.
	Minimum Average Displacement Error (minADE)	$\mathrm{minADE}=\min\left(\frac{\sum_{i=1}^{N}\sum_{t=t_{obs}+1}^{t_{pred}}{∥Y_{i}^{t}-{\hat{Y}}_{i,j}^{t}∥}_{2}}{N\times (t_{pred}-t_{obs})}\right)$, where $Y_{i}^{t}$ represents the actual position of pedestrian *i* at time *t*, and ${\hat{Y}}_{i,j}^{t}$ denotes the *j*-th predicted position of pedestrian *i* at time *t*, where *j* indexes different trajectory predictions for pedestrian. *i*.
	Minimum Final Displacement Error (minFDE)	$\mathrm{minFDE}=\min\left(\frac{\sum_{i=1}^{N}{∥Y_{i}^{t_{pred}}-{\hat{Y}}_{i,j}^{t_{pred}}∥}_{2}}{N}\right)$, dividing by *N* normalizes the sum, giving the average Minimum Final Displacement Error across all individuals.
Probabilistic Distribution Metrics	Negative Log-Likelihood (NLL)	$\mathrm{NLL}=-\sum_{i=1}^{n}y_{i}\log\left({\hat{y}}_{n}\right)$, where NLL stands for negative log-likelihood, measuring the likelihood of observing the given outcomes under the model’s predicted probability distribution. $y_{i}$ denotes a binary indicator of the correctness of predicting the data sample in class *i*, ${\hat{y}}_{n}$ is the predicted probability of the data sample belonging to class *i*, and *n* is the total number of classes.
	Kernel Density Estimation NLL (KDE-NLL)	$\mathrm{KDE}-\mathrm{NLL}=-\frac{\sum_{i=1}^{n}\mathrm{NLL}\left(P\left(\hat{y},i\right),P\left(y,i\right)\right)}{n}$, where the kernel density estimation negative log-likelihood evaluates the match between predicted and observed probability distributions. $P(\hat{y},i)$ is the predicted probability distribution at the *i*-th data point, and P(y,i) is the true probability distribution at the *i*-th data point.

**Table 9:** Method performance comparison.

Method	ETH (ADE/FDE)	HOTEL (ADE/FDE)	UNIV (ADE/FDE)	ZARA1 (ADE/FDE)	ZARA2 (ADE/FDE)	AVG (ADE/FDE)
Social-LSTM	0.46/0.95	0.15/0.29	0.22/0.45	0.29/0.48	0.22/0.67	0.27/0.57
SR-LSTM	0.63/1.25	0.37/0.74	0.51/1.10	0.41/0.90	0.32/0.70	0.45/0.94
Trajectron++	0.71/1.68	0.22/0.46	0.41/1.07	0.30/0.77	0.23/0.59	0.37/0.91
Social-GAN	0.81/1.52	0.72/1.61	0.60/1.26	0.34/0.69	0.42/0.84	0.58/1.18
CGNS	0.62/1.40	0.70/0.93	0.48/1.22	0.32/0.59	0.35/0.71	0.49/0.97
Social-BiGAT	0.69/1.29	0.49/1.01	0.55/1.32	0.30/0.62	0.36/0.75	0.48/1.00
Sophie	0.70/1.43	0.76/1.67	0.54/1.24	0.30/0.63	0.38/0.78	0.54/1.15
STGAN	0.65/1.12	0.35/0.66	0.52/1.10	0.34/0.69	0.29/0.60	0.43/0.83
Social-STGCNN	0.64/1.11	0.49/0.85	0.44/0.79	0.34/0.53	0.30/0.48	0.44/0.75
Attention-GCNN	0.68/1.22	0.31/0.41	0.39/0.69	0.34/0.55	0.28/0.44	0.40/0.66
AVGCN	0.62/1.06	0.31/0.58	0.55/1.20	0.33/0.70	0.27/0.58	0.41/0.82
PTP-STGCN	0.63/1.04	0.34/0.45	0.48/0.87	0.37/0.61	0.30/0.46	0.42/0.68
RSBG	0.80/1.53	0.33/0.64	0.59/1.25	0.40/0.86	0.30/0.65	0.48/0.99
GraphTCN	0.39/0.71	0.21/0.44	0.33/0.66	0.21/0.42	0.17/0.43	0.26/0.51
SGCN	0.63/1.03	0.32/0.55	0.37/0.70	0.29/0.53	0.25/0.45	0.37/0.65
Grouptron	0.70/1.56	0.21/0.46	0.38/0.97	0.30/0.76	0.22/0.56	0.36/0.86
PECNet	0.54/0.87	0.18/0.24	0.35/0.60	0.22/0.39	0.17/0.30	0.29/0.48
VDRGCN	0.62/0.81	0.27/0.37	0.38/0.58	0.29/0.42	0.21/0.32	0.35/0.50
DGCN+STDec	0.28/0.62	0.15/0.31	0.38/0.88	0.27/0.60	0.24/0.57	0.26/0.59
STAR	0.56/1.11	0.26/0.50	0.52/1.15	0.41/0.90	0.31/0.71	0.41/0.87
AgentFormer	0.26/0.39	0.11/0.14	0.26/0.46	0.15/0.23	0.14/0.24	0.18/0.29
MID	0.39/0.66	0.13/0.22	0.22/0.45	0.17/0.30	0.13/0.27	0.21/0.38
SGTN	0.40/0.59	0.14/0.16	0.16/0.58	0.17/0.29	0.14/0.17	0.20/0.28
AFC-RNN	0.37/0.56	0.12/0.19	0.20/0.36	0.16/0.30	0.12/0.21	0.19/0.32
DERGCN	0.48/0.73	0.25/0.45	0.49/1.05	0.30/0.60	0.28/0.59	0.36/0.68
SIAT	0.43/0.61	0.12/0.17	0.24/0.43	0.18/0.33	0.14/0.25	0.22/0.36
ASTRA	0.47/0.82	0.29/0.56	0.55/1.00	0.34/0.71	0.24/0.41	0.38/0.70

## Data Availability

The ETH public dataset in this paper is available at 5 November 2025 https://data.vision.ee.ethz.ch/cvl/aem/ewap_dataset_full.tgz. The UCY public dataset in this paper is available at 5 November 2025 https://graphics.cs.ucy.ac.cy/research/downloads/crowd-data.

## References

[1] Luber M., Stork J.A., Tipaldi G.D., Arras K.O. People tracking with human motion predictions from social forces. Proceedings of the 2010 IEEE International Conference on Robotics and Automation.

[2] Dragan A.D., Ratliff N.D., Srinivasa S.S. Manipulation planning with goal sets using constrained trajectory optimization. Proceedings of the 2011 IEEE International Conference on Robotics and Automation.

[3] Kuderer M., Kretzschmar H., Sprunk C., Burgard W. (2012). Feature-based prediction of trajectories for socially compliant navigation. Robotics: Science and Systems VIII.

[4] Mainprice J., Hayne R., Berenson D. (2016). Goal set inverse optimal control and iterative replanning for predicting human reaching motions in shared workspaces. IEEE Trans. Robot..

[5] Trautman P., Krause A. Unfreezing the robot: Navigation in dense, interacting crowds. Proceedings of the 2010 IEEE/RSJ International Conference on Intelligent Robots and Systems.

[6] Ziebart B.D., Ratliff N., Gallagher G., Mertz C., Peterson K., Bagnell J.A., Hebert M., Dey A.K., Srinivasa S. Planning-based prediction for pedestrians. Proceedings of the 2009 IEEE/RSJ International Conference on Intelligent Robots and Systems.

[7] Rehder E., Wirth F., Lauer M., Stiller C. Pedestrian prediction by planning using deep neural networks. Proceedings of the 2018 IEEE International Conference on Robotics and Automation (ICRA).

[8] Toroyan T. (2009). Global status report on road safety. Inj. Prev..

[9] Helbing D., Molnar P. (1995). Social force model for pedestrian dynamics. Phys. Rev. E.

[10] Pellegrini S., Ess A., Van Gool L. (2010). Improving data association by joint modeling of pedestrian trajectories and groupings. Proceedings of the Computer Vision–ECCV 2010: 11th European Conference on Computer Vision.

[11] Koppula H.S., Saxena A. (2015). Anticipating human activities using object affordances for reactive robotic response. IEEE Trans. Pattern Anal. Mach. Intell..

[12] Rudenko A., Palmieri L., Herman M., Kitani K.M., Gavrila D.M., Arras K.O. (2020). Human motion trajectory prediction: A survey. Int. J. Robot. Res..

[13] Xu Y., Piao Z., Gao S. Encoding crowd interaction with deep neural network for pedestrian trajectory prediction. Proceedings of the IEEE Conference on Computer Vision and Pattern Recognition.

[14] Alahi A., Goel K., Ramanathan V., Robicquet A., Fei-Fei L., Savarese S. Social lstm: Human trajectory prediction in crowded spaces. Proceedings of the IEEE Conference on Computer Vision and Pattern Recognition.

[15] Chen Z. (2003). Bayesian filtering: From Kalman filters to particle filters, and beyond. Statistics.

[16] Lauritzen S.L., Spiegelhalter D.J. (1988). Local computations with probabilities on graphical structures and their application to expert systems. J. R. Stat. Soc. Ser. B Methodol..

[17] Ferguson S., Luders B., Grande R.C., How J.P. (2015). Real-time predictive modeling and robust avoidance of pedestrians with uncertain, changing intentions. Algorithmic Foundations of Robotics XI: Selected Contributions of the Eleventh International Workshop on the Algorithmic Foundations of Robotics.

[18] Ghahramani Z., Jordan M. (1995). Factorial hidden Markov models. Adv. Neural Inf. Process. Syst..

[19] Kooij J.F.P., Schneider N., Flohr F., Gavrila D.M. (2014). Context-based pedestrian path prediction. Proceedings of the Computer Vision–ECCV 2014: 13th European Conference.

[20] Kalman R.E. (1960). A new approach to linear filtering and prediction problems. J. Basic Eng. Mar..

[21] Nelder J.A., Wedderburn R.W. (1972). Generalized linear models. J. R. Stat. Soc. Ser. A Stat. Soc..

[22] Tay M.K.C., Laugier C. (2008). Modelling smooth paths using gaussian processes. Proceedings of the Field and Service Robotics: Results of the 6th International Conference.

[23] Wang J.M., Fleet D.J., Hertzmann A. (2007). Gaussian process dynamical models for human motion. IEEE Trans. Pattern Anal. Mach. Intell..

[24] Williams C.K. (1998). Prediction with Gaussian processes: From linear regression to linear prediction and beyond. Learning in Graphical Models.

[25] Quinonero-Candela J., Rasmussen C.E. (2005). A unifying view of sparse approximate Gaussian process regression. J. Mach. Learn. Res..

[26] Rasmussen C.E. (2003). Gaussian processes in machine learning. Summer School on Machine Learning.

[27] Akaike H. (1969). Fitting autoregreesive models for prediction. Selected Papers of Hirotugu Akaike.

[28] Jenkins G.M., Priestley M. (1957). The spectral analysis of time-series. J. R. Stat. Soc. Ser. B Methodol..

[29] Rumelhart D.E., Hinton G.E., Williams R.J. (1986). Learning representations by back-propagating errors. Nature.

[30] Graves A., Jaitly N. Towards end-to-end speech recognition with recurrent neural networks. Proceedings of the International Conference on Machine Learning.

[31] Chorowski J., Bahdanau D., Cho K., Bengio Y. (2014). End-to-end continuous speech recognition using attention-based recurrent nn: First results. arXiv.

[32] Chung J., Kastner K., Dinh L., Goel K., Courville A.C., Bengio Y. (2015). A recurrent latent variable model for sequential data. Adv. Neural Inf. Process. Syst..

[33] Graves A., Mohamed A.r., Hinton G. Speech recognition with deep recurrent neural networks. Proceedings of the 2013 IEEE International Conference on Acoustics, Speech and Signal Processing.

[34] Vinyals O., Toshev A., Bengio S., Erhan D. Show and tell: A neural image caption generator. Proceedings of the IEEE Conference on Computer Vision and Pattern Recognition.

[35] Karpathy A., Joulin A., Fei-Fei L.F. (2014). Deep fragment embeddings for bidirectional image sentence mapping. Adv. Neural Inf. Process. Syst..

[36] Yoo D., Park S., Lee J.Y., Paek A.S., So Kweon I. Attentionnet: Aggregating weak directions for accurate object detection. Proceedings of the IEEE International Conference on Computer Vision.

[37] Xu K. (2015). Show, attend and tell: Neural image caption generation with visual attention. arXiv.

[38] Donahue J., Anne Hendricks L., Guadarrama S., Rohrbach M., Venugopalan S., Saenko K., Darrell T. Long-term recurrent convolutional networks for visual recognition and description. Proceedings of the IEEE Conference on Computer Vision and Pattern Recognition.

[39] Bahdanau D. (2014). Neural machine translation by jointly learning to align and translate. arXiv.

[40] Sutskever I. (2014). Sequence to Sequence Learning with Neural Networks. arXiv.

[41] Cho K., Van Merriënboer B., Gulcehre C., Bahdanau D., Bougares F., Schwenk H., Bengio Y. (2014). Learning phrase representations using RNN encoder-decoder for statistical machine translation. arXiv.

[42] Zheng S., Jayasumana S., Romera-Paredes B., Vineet V., Su Z., Du D., Huang C., Torr P.H. Conditional random fields as recurrent neural networks. Proceedings of the IEEE International Conference on Computer Vision.

[43] Cao C., Liu X., Yang Y., Yu Y., Wang J., Wang Z., Huang Y., Wang L., Huang C., Xu W. Look and think twice: Capturing top-down visual attention with feedback convolutional neural networks. Proceedings of the IEEE International Conference on Computer Vision.

[44] Gregor K., Danihelka I., Graves A., Rezende D., Wierstra D. Draw: A recurrent neural network for image generation. Proceedings of the International Conference on Machine Learning.

[45] Xiao T., Xu Y., Yang K., Zhang J., Peng Y., Zhang Z. The application of two-level attention models in deep convolutional neural network for fine-grained image classification. Proceedings of the IEEE Conference on Computer Vision and Pattern Recognition.

[46] Yue-Hei Ng J., Hausknecht M., Vijayanarasimhan S., Vinyals O., Monga R., Toderici G. Beyond short snippets: Deep networks for video classification. Proceedings of the IEEE Conference on Computer Vision and Pattern Recognition.

[47] Pinheiro P., Collobert R. Recurrent convolutional neural networks for scene labeling. Proceedings of the International Conference on Machine Learning.

[48] Hochreiter S. (1997). Long Short-term Memory. Neural Computation.

[49] Chung J., Gulcehre C., Cho K., Bengio Y. (2014). Empirical evaluation of gated recurrent neural networks on sequence modeling. arXiv.

[50] Goodfellow I., Pouget-Abadie J., Mirza M., Xu B., Warde-Farley D., Ozair S., Courville A., Bengio Y. (2014). Generative adversarial nets. Adv. Neural Inf. Process. Syst..

[51] Kingma D.P. (2013). Auto-encoding variational bayes. arXiv.

[52] Kipf T.N., Welling M. (2016). Semi-supervised classification with graph convolutional networks. arXiv.

[53] Vaswani A. (2017). Attention is all you need. Adv. Neural Inf. Process. Syst..

[54] Liu Q., Wu S., Wang L., Tan T. Predicting the next location: A recurrent model with spatial and temporal contexts. Proceedings of the AAAI Conference on Artificial Intelligence.

[55] Lee N., Choi W., Vernaza P., Choy C.B., Torr P.H., Chandraker M. Desire: Distant future prediction in dynamic scenes with interacting agents. Proceedings of the IEEE Conference on Computer Vision and Pattern Recognition.

[56] Su H., Zhu J., Dong Y., Zhang B. Forecast the Plausible Paths in Crowd Scenes. Proceedings of the IJCAI.

[57] Hasan I., Setti F., Tsesmelis T., Del Bue A., Galasso F., Cristani M. Mx-lstm: Mixing tracklets and vislets to jointly forecast trajectories and head poses. Proceedings of the IEEE Conference on Computer Vision and Pattern Recognition.

[58] Vemula A., Muelling K., Oh J. Social attention: Modeling attention in human crowds. Proceedings of the 2018 IEEE International Conference on Robotics and Automation (ICRA).

[59] Fernando T., Denman S., Sridharan S., Fookes C. (2018). Soft+ hardwired attention: An lstm framework for human trajectory prediction and abnormal event detection. Neural Netw..

[60] Xue H., Huynh D.Q., Reynolds M. SS-LSTM: A hierarchical LSTM model for pedestrian trajectory prediction. Proceedings of the 2018 IEEE Winter Conference on Applications of Computer Vision (WACV).

[61] Zhang P., Ouyang W., Zhang P., Xue J., Zheng N. Sr-lstm: State refinement for lstm towards pedestrian trajectory prediction. Proceedings of the IEEE/CVF Conference on Computer Vision and Pattern Recognition.

[62] Lisotto M., Coscia P., Ballan L. Social and scene-aware trajectory prediction in crowded spaces. Proceedings of the IEEE/CVF International Conference on Computer Vision Workshops.

[63] Song X., Chen K., Li X., Sun J., Hou B., Cui Y., Zhang B., Xiong G., Wang Z. (2020). Pedestrian trajectory prediction based on deep convolutional LSTM network. IEEE Trans. Intell. Transp. Syst..

[64] Salzmann T., Ivanovic B., Chakravarty P., Pavone M. (2020). Trajectron++: Dynamically-feasible trajectory forecasting with heterogeneous data. Proceedings of the Computer Vision–ECCV 2020: 16th European Conference.

[65] Xu Y., Fu Y. Adapting to length shift: Flexilength network for trajectory prediction. Proceedings of the IEEE/CVF Conference on Computer Vision and Pattern Recognition.

[66] Tang X., Kan M., Shan S., Ji Z., Bai J., Chen X. Hpnet: Dynamic trajectory forecasting with historical prediction attention. Proceedings of the IEEE/CVF Conference on Computer Vision and Pattern Recognition.

[67] Dong Y., Wang L., Zhou S., Tang W., Hua G., Sun C. (2025). AFC-RNN: Adaptive Forgetting-Controlled Recurrent Neural Network for Pedestrian Trajectory Prediction. IEEE Trans. Pattern Anal. Mach. Intell..

[68] Zamboni S., Kefato Z.T., Girdzijauskas S., Norén C., Dal Col L. (2022). Pedestrian trajectory prediction with convolutional neural networks. Pattern Recognit..

[69] Wang D., Liu H., Wang N., Wang Y., Wang H., McLoone S. (2022). SEEM: A sequence entropy energy-based model for pedestrian trajectory all-then-one prediction. IEEE Trans. Pattern Anal. Mach. Intell..

[70] Khel M.H.K., Greaney P., McAfee M., Moffett S., Meehan K. Pedestrian Trajectory Prediction using BiLSTM with Spatial-Temporal Attention and Sparse Motion Fields. Proceedings of the 2023 34th Irish Signals and Systems Conference (ISSC).

[71] Yang J., Chen Y., Du S., Chen B., Principe J.C. (2024). IA-LSTM: Interaction-aware LSTM for pedestrian trajectory prediction. IEEE Trans. Cybern..

[72] Arjovsky M., Bottou L. (2017). Towards principled methods for training generative adversarial networks. arXiv.

[73] Salimans T., Goodfellow I., Zaremba W., Cheung V., Radford A., Chen X. (2016). Improved techniques for training gans. Adv. Neural Inf. Process. Syst..

[74] Gupta A., Johnson J., Fei-Fei L., Savarese S., Alahi A. Social gan: Socially acceptable trajectories with generative adversarial networks. Proceedings of the IEEE Conference on Computer Vision and Pattern Recognition.

[75] Li J., Ma H., Tomizuka M. Conditional generative neural system for probabilistic trajectory prediction. Proceedings of the 2019 IEEE/RSJ International Conference on Intelligent Robots and Systems (IROS).

[76] Kothari P., Alahi A. Human trajectory prediction using adversarial loss. Proceedings of the 19th Swiss Transport Research Conference.

[77] Kosaraju V., Sadeghian A., Martín-Martín R., Reid I., Rezatofighi H., Savarese S. (2019). Social-bigat: Multimodal trajectory forecasting using bicycle-gan and graph attention networks. Adv. Neural Inf. Process. Syst..

[78] Amirian J., Hayet J.B., Pettré J. Social ways: Learning multi-modal distributions of pedestrian trajectories with gans. Proceedings of the IEEE/CVF Conference on Computer Vision and Pattern Recognition Workshops.

[79] Sadeghian A., Kosaraju V., Sadeghian A., Hirose N., Rezatofighi H., Savarese S. Sophie: An attentive gan for predicting paths compliant to social and physical constraints. Proceedings of the IEEE/CVF Conference on Computer Vision and Pattern Recognition.

[80] Sun H., Zhao Z., He Z. Reciprocal learning networks for human trajectory prediction. 2020 IEEE. Proceedings of the CVF Conference on Computer Vision and Pattern Recognition.

[81] Zou X., Sun B., Zhao D., Zhu Z., Zhao J., He Y. (2020). Multi-modal pedestrian trajectory prediction for edge agents based on spatial-temporal graph. IEEE Access.

[82] Zhong J., Sun H., Cao W., He Z. (2020). Pedestrian motion trajectory prediction with stereo-based 3D deep pose estimation and trajectory learning. IEEE Access.

[83] Yang B., He C., Wang P., Chan C.y., Liu X., Chen Y. (2020). TPPO: A novel trajectory predictor with pseudo oracle. arXiv.

[84] Zhou Z., Huang G., Su Z., Li Y., Hua W. (2022). Dynamic attention-based CVAE-GAN for pedestrian trajectory prediction. IEEE Robot. Autom. Lett..

[85] An H., Liu M., Wang X., Zhang W., Gong J. (2024). Multi Attention Generative Adversarial Network for Pedestrian Trajectory Prediction Based on Spatial Gridding. Automot. Innov..

[86] Li Y., Zhang C., Zhou J., Zhou S. (2024). POI-GAN: A pedestrian trajectory prediction method for service scenarios. IEEE Access.

[87] LeCun Y., Bottou L., Bengio Y., Haffner P. (1998). Gradient-based learning applied to document recognition. Proc. IEEE.

[88] Yi S., Li H., Wang X. (2016). Pedestrian behavior understanding and prediction with deep neural networks. Proceedings of the Computer Vision–ECCV 2016: 14th European Conference.

[89] Scarselli F., Gori M., Tsoi A.C., Hagenbuchner M., Monfardini G. (2008). The graph neural network model. IEEE Trans. Neural Netw..

[90] Abdullah M., He J., Wang K. (2022). Weather-aware fiber-wireless traffic prediction using graph convolutional networks. IEEE Access.

[91] Zhang L., She Q., Guo P. (2019). Stochastic trajectory prediction with social graph network. arXiv.

[92] Mohamed A., Qian K., Elhoseiny M., Claudel C. Social-stgcnn: A social spatio-temporal graph convolutional neural network for human trajectory prediction. Proceedings of the IEEE/CVF Conference on Computer Vision and Pattern Recognition.

[93] Li K., Eiffert S., Shan M., Gomez-Donoso F., Worrall S., Nebot E. Attentional-GCNN: Adaptive pedestrian trajectory prediction towards generic autonomous vehicle use cases. Proceedings of the 2021 IEEE International Conference on Robotics and Automation (ICRA).

[94] Liu C., Chen Y., Liu M., Shi B.E. AVGCN: Trajectory prediction using graph convolutional networks guided by human attention. Proceedings of the 2021 IEEE International Conference on Robotics and Automation (ICRA).

[95] Lian J., Ren W., Li L., Zhou Y., Zhou B. (2023). Ptp-stgcn: Pedestrian trajectory prediction based on a spatio-temporal graph convolutional neural network. Appl. Intell..

[96] Wang C., Cai S., Tan G. Graphtcn: Spatio-temporal interaction modeling for human trajectory prediction. Proceedings of the IEEE/CVF Winter Conference on Applications of Computer Vision.

[97] Sun J., Jiang Q., Lu C. Recursive social behavior graph for trajectory prediction. Proceedings of the IEEE/CVF Conference on Computer Vision and Pattern Recognition.

[98] Rainbow B.A., Men Q., Shum H.P. Semantics-STGCNN: A semantics-guided spatial-temporal graph convolutional network for multi-class trajectory prediction. Proceedings of the 2021 IEEE International Conference on Systems, Man, and Cybernetics (SMC).

[99] Shi L., Wang L., Long C., Zhou S., Zhou M., Niu Z., Hua G. SGCN: Sparse graph convolution network for pedestrian trajectory prediction. Proceedings of the IEEE/CVF Conference on Computer Vision and Pattern Recognition.

[100] Zhou R., Zhou H., Gao H., Tomizuka M., Li J., Xu Z. Grouptron: Dynamic multi-scale graph convolutional networks for group-aware dense crowd trajectory forecasting. Proceedings of the 2022 International Conference on Robotics and Automation (ICRA).

[101] Su Y., Du J., Li Y., Li X., Liang R., Hua Z., Zhou J. (2022). Trajectory forecasting based on prior-aware directed graph convolutional neural network. IEEE Trans. Intell. Transp. Syst..

[102] Bhujel N., Yau W.Y. (2023). Disentangling crowd interactions for pedestrians trajectory prediction. IEEE Robot. Autom. Lett..

[103] Mi J., Zhang X., Zeng H., Wang L. (2024). DERGCN: Dynamic-evolving graph convolutional networks for human trajectory prediction. Neurocomputing.

[104] Feng A., Gong J., Wang N. (2024). Pedestrian Trajectory Prediction Algorithm Based on Graph Convolution and Convolution. J. Northeast. Univ. Natural Sci..

[105] Yu C., Ma X., Ren J., Zhao H., Yi S. (2020). Spatio-temporal graph transformer networks for pedestrian trajectory prediction. Proceedings of the Computer Vision–ECCV 2020: 16th European Conference.

[106] Yin Z., Liu R., Xiong Z., Yuan Z. Multimodal Transformer Networks for Pedestrian Trajectory Prediction. Proceedings of the IJCAI.

[107] Yuan Y., Weng X., Ou Y., Kitani K.M. Agentformer: Agent-aware transformers for socio-temporal multi-agent forecasting. Proceedings of the IEEE/CVF International Conference on Computer Vision.

[108] Li L., Pagnucco M., Song Y. Graph-based spatial transformer with memory replay for multi-future pedestrian trajectory prediction. Proceedings of the IEEE/CVF Conference on Computer Vision and Pattern Recognition.

[109] Gu T., Chen G., Li J., Lin C., Rao Y., Zhou J., Lu J. Stochastic trajectory prediction via motion indeterminacy diffusion. Proceedings of the IEEE/CVF Conference on Computer Vision and Pattern Recognition.

[110] Liu Y., Yao L., Li B., Wang X., Sammut C. Social graph transformer networks for pedestrian trajectory prediction in complex social scenarios. Proceedings of the 31st ACM International Conference on Information & Knowledge Management.

[111] Shi L., Wang L., Zhou S., Hua G. Trajectory unified transformer for pedestrian trajectory prediction. Proceedings of the IEEE/CVF International Conference on Computer Vision.

[112] Tamaru R., Li P., Ran B. (2024). Enhancing Pedestrian Trajectory Prediction with Crowd Trip Information. arXiv.

[113] Lin X., Liang T., Lai J., Hu J.F. (2024). Progressive pretext task learning for human trajectory prediction. Proceedings of the European Conference on Computer Vision.

[114] Wang C., Wang J., Gao W., Guo L. (2025). SIAT: Pedestrian trajectory prediction via social interaction-aware transforme. Complex Intell. Syst..

[115] Teeti I., Thomas A., Monga M., Kumar S., Singh U., Bradley A., Banerjee B., Cuzzolin F. (2025). ASTRA: A Scene-aware TRAnsformer-based model for trajectory prediction. arXiv.

[116] Liang R., Li Y., Zhou J., Li X. (2023). STGlow: A flow-based generative framework with dual-graphormer for pedestrian trajectory prediction. IEEE Trans. Neural Netw. Learn. Syst..

[117] Mangalam K., Girase H., Agarwal S., Lee K.H., Adeli E., Malik J., Gaidon A. (2020). It is not the journey but the destination: Endpoint conditioned trajectory prediction. Proceedings of the Computer Vision–ECCV 2020: 16th European Conference.

[118] Mangalam K., An Y., Girase H., Malik J. From goals, waypoints & paths to long term human trajectory forecasting. Proceedings of the IEEE/CVF International Conference on Computer Vision.

[119] Gu J., Sun C., Zhao H. Densetnt: End-to-end trajectory prediction from dense goal sets. Proceedings of the IEEE/CVF International Conference on Computer Vision.

[120] Guo K., Liu W., Pan J. End-to-end trajectory distribution prediction based on occupancy grid maps. Proceedings of the IEEE/CVF Conference on Computer Vision and Pattern Recognition.

[121] Korbmacher R., Tordeux A. (2022). Review of pedestrian trajectory prediction methods: Comparing deep learning and knowledge-based approaches. IEEE Trans. Intell. Transp. Syst..

[122] Pellegrini S., Ess A., Schindler K., Van Gool L. You’ll never walk alone: Modeling social behavior for multi-target tracking. Proceedings of the 2009 IEEE 12th International Conference on Computer Vision.

[123] Lerner A., Chrysanthou Y., Lischinski D. (2007). Crowds by example. Computer Graphics Forum.

[124] Robicquet A., Sadeghian A., Alahi A., Savarese S. (2016). Learning social etiquette: Human trajectory understanding in crowded scenes. Proceedings of the Computer Vision–ECCV 2016: 14th European Conference.

[125] Lian J., Wang X. (2021). Pedestrian trajectory prediction based on human-vehicle interaction. China J. Highw. Transp..

[126] Caesar H., Bankiti V., Lang A.H., Vora S., Liong V.E., Xu Q., Krishnan A., Pan Y., Baldan G., Beijbom O. nuscenes: A multimodal dataset for autonomous driving. Proceedings of the IEEE/CVF Conference on Computer Vision and Pattern Recognition.

[127] Chang M.F., Lambert J., Sangkloy P., Singh J., Bak S., Hartnett A., Wang D., Carr P., Lucey S., Ramanan D. Argoverse: 3d tracking and forecasting with rich maps. Proceedings of the IEEE/CVF conference on Computer Vision and Pattern Recognition.

[128] Rasouli A., Kotseruba I., Kunic T., Tsotsos J.K. Pie: A large-scale dataset and models for pedestrian intention estimation and trajectory prediction. Proceedings of the IEEE/CVF conference on Computer Vision and Pattern Recognition.

[129] Kothari P., Kreiss S., Alahi A. (2021). Human trajectory forecasting in crowds: A deep learning perspective. IEEE Trans. Intell. Transp. Syst..

[130] Deo N., Trivedi M.M. Multi-modal trajectory prediction of surrounding vehicles with maneuver based lstms. Proceedings of the 2018 IEEE Intelligent Vehicles Symposium (IV).

[131] Koutrintzes D., Spyrou E., Mathe E., Mylonas P. (2023). A multimodal fusion approach for human activity recognition. Int. J. Neural Syst..

[132] Obaid L., Hamad K., Al-Ruzouq R., Dabous S.A., Ismail K. (2025). Automating the estimation of turning movement rates at multilane roundabouts using unmanned aerial vehicles and deep learning. Green Energy Intell. Transp..

[133] Li Z., Gong C., Lin Y., Li G., Wang X., Lu C., Wang M., Chen S., Gong J. (2023). Continual driver behaviour learning for connected vehicles and intelligent transportation systems: Framework, survey and challenges. Green Energy Intell. Transp..

